# Combined Substitution of Fishmeal and Fish Oil With Black Soldier Fly Larval Meal and Corn Oil: Effects on Growth, Hematology, Hematobiochemical, Amino and Fatty Acid Composition, and Fillet Nutritional Quality of Hybrid African Catfish *Clarias gariepinus* × *Heterobranchus longifilis*

**DOI:** 10.1155/anu/4880013

**Published:** 2026-03-30

**Authors:** Christopher Teye-Gaga, Péter István Molnár, Attila Kertész, John Kiguru Maina, Elshafia Ali Hamid Mohammed, Gabriella Gulyás, Levente Czeglédi, Milán Fehér, Péter Bársony

**Affiliations:** ^1^ Faculty of Agricultural and Food Sciences and Environmental Management, Institute of Animal Science, Biotechnology and Nature, Department of Animal Science, University of Debrecen, 138 Böszörményi Street, Debrecen, 4032, Hungary, unideb.hu; ^2^ Doctoral School of Animal Science, University of Debrecen, 138 Böszörményi Street, Debrecen, 4032, Hungary, unideb.hu; ^3^ Faculty of Agricultural and Food Sciences and Environmental Management, Institute of Animal Science, Biotechnology and Nature, Department of Nutrition Physiology, University of Debrecen, 138 Böszörményi Street, Debrecen, 4032, Hungary, unideb.hu

**Keywords:** alternative ingredients, health status, muscle composition, production performance, sustainable aquaculture

## Abstract

This study aimed to evaluate growth performance, amino acid and fatty acid composition of fillets, and blood biochemistry of hybrid catfish (*Heteroclarias*) cultured on Black Soldier Fly (BSF), *Hermetia illucens* larval‐based diets. The experiment was conducted in a recirculating system, circular poly tanks (350 L), in a completely randomized design. Four isonitrogenous (400 g kg^−1^ crude protein) and isolipidic (140 g kg^−1^ crude fat) diets were formulated in which fishmeal (400 g kg^−1^) was replaced at 0%, 25%, 50%, and 75% with defatted BSF larval meal and fish oil was completely replaced with corn oil in the test diets. 180 hybrid African catfish (12 weeks post‐hatching) with an initial body weight of 200 ± 25 g were randomly distributed in the 12 experimental tanks (15 fish per tank, 45 fish per treatment) and were hand‐fed at 3% body weight for 8 weeks. The findings showed that replacing 50% of fishmeal with BSF meal resulted in the highest growth performance (final weight, weight gain, specific growth rate [SGR], thermal growth coefficient [TGC]). However, at 75% level, growth performance and nutrient utilization (FCR, PER) significantly decline (*p*  < 0.05). The dietary modification had no significant impact (*p*  > 0.05) on organosomatic indices, proximate composition, amino acid profile, deposition, or retention of arachidonic acid (ARA), eicosapentaenoic acid (EPA), docosahexaenoic acid (DHA), and sum n‐3 polyunsaturated fatty acids (PUFA) in fish fillets. There were no significant differences in the hematological parameters (*p*  > 0.05) across all treatments. Except for a reduction in globulin and cholesterol levels, all the plasma metabolites, including alanine aminotransferase (ALT) and aspartate aminotransferase (AST) levels, remained stable (*p*  > 0.05). Overall, the findings of this study suggest that BSF larval meal may partially replace dietary fishmeal up to 50% (200 g kg^−1^), and corn oil may completely replace fish oil in a practical diet for hybrid African catfish without exerting adverse impacts on growth, feed conversion efficiency, fillet quality, health status, and physiological well‐being.

## 1. Introduction

Fish is an essential source of protein for humanity, accounting for at least 15% of the world’s animal protein intake. Over 3.2 billion people, mainly in low‐income countries, rely heavily on fish to meet their nutritional needs, reflecting its affordability, availability and accessibility [1]. Currently, as the leading source of seafood supply, aquaculture continues to play a crucial role in meeting demands for animal protein. Fishmeal and fish oil are the optimal protein and lipid components in fish feed formulation. However, the rising cost of these ingredients, along with concerns about their sustainability and ecological impacts on fisheries, has led to efforts to evaluate a wide range of relatively low‐cost, sustainable ingredients that could partially or entirely replace fishmeal and fish oil [1, 2].

In recent decades, various insects (particularly larvae) have been increasingly utilized as attractive non‐conventional protein and fat ingredient options in animal feed formulation and utilization [3–5]. Aquatic insects are a natural diet for fish. Insects are highly nutritious, fast‐growing, and easy to produce using low‐value organic products, requiring minimal water and land, and leaving no environmental carbon footprint [6–8]. Among the eight insects approved for use in aquafeed by the European Union Commission (EU regulation 2017/893‐24/05/2017 and 2021/1925), the Black Soldier Fly (BSF) larvae are the most studied [9].

The nutritional composition of the larvae varies depending on their diet, life stage, rearing conditions and processing method [10, 11]. The larvae fed on poultry manure contained about 42% protein and 35% fats (dry matter basis) [8, 12]. The process of defatting may reduce the lipid content (to 9% or less) and consequently increase the protein content to ~60% DM [13–15]. BSF larvae have a well‐balanced amino acid profile similar to that of fishmeal and are a rich source of vitamins and essential minerals [4, 16, 17]. Though rich in fat, the larvae have a limited ability to bioconvert short‐chain polyunsaturated fatty acid (SC‐PUFA) to long‐chain (LC)‐PUFA, resulting in generally low levels of n‐3 LC‐PUFA [18, 19]. Nonetheless, numerous studies have demonstrated that incorporating BSF larval meal into formulated diets promotes healthy fish growth, including that of African catfish *C. gariepinus* [20–22].

Corn oil is a highly dense, easily digestible oil rich in n‐6 PUFA, particularly linoleic acid (LA), which constitutes about 58%–62% of the total fatty acid [23–25]. It contains tocopherols (Vitamin E) and phytosterols, which act as antioxidants and slow lipid peroxidation [24, 26, 27]. Compared to fish oil, corn oil is a cost‐effective and sustainable lipid source. Although it is low in n‐3 PUFA, some studies have shown that corn oil, either singly or as a blend of vegetable oils, may partially replace fish oil in diets for freshwater aquaculture species without negatively affecting production performance, as in Nile tilapia *Oreochromis niloticus* [23], grouper *Epinephelus malabaricus* [28], the gibel carp *Carassius auratus gibelio* [29], and tench *Tinca tinca* [30]. While vegetable oils such as soybean, sunflower, rapeseed, and linseed are commonly used in aquafeed, corn oil has been less extensively studied. In addition to its nutritional benefits, its relatively lower susceptibility to rancidity was another reason for its inclusion in this research.

The African catfish, *C. gariepinus* (Clariidae), is one of the most economically important aquaculture fish species globally due to its high fecundity, good feed conversion, disease resistance, high fillet quality, and good taste [31–33]. Between 2010 and 2020, global Clarias catfish production increased more than threefold, from 343,300 metric tonnes to 1.25 million metric tonnes, accounting for 2.5% of global inland finfish aquaculture production [34].

In recent decades, many catfish‐producing nations (such as Hungary, which is the largest producer of African catfish in Europe) have been offering hybrid catfish, *Heteroclarias*, produced in hatcheries using oocytes from *Clarias gariepinus* and milt from *Heterobranchus longifilis* or vice versa. The resulting fry typically grows faster than the parental species. However, this hybrid has been reported to exhibit aggressive behavior, partly due to large variations in body sizes as they develop [35, 36].

Previous research has demonstrated that fish fillets produced from insect‐based diets contain low levels of valuable omega‐3 LC‐PUFA (n‐3 LC‐PUFA), which is a major drawback as it may have implications for both fish species and human nutrition [37–40].

Although numerous studies have explored the use of BSF meal as a partial or complete replacement for fishmeal in various purebred fish species, including *C. gariepinus*, most focused on single‐ingredient substitution while maintaining fish oil as the primary lipid source. In contrast, the present study evaluates a combined fishmeal‐fish oil replacement in hybrid catfish, *Heteroclarias*. This approach reflects a more realistic, sustainable and economically viable strategy for feed formulation.

Therefore, the objective of this study was to quantitatively assess the species‐specific response of *heteroclarias* to combined dietary substitutions of fishmeal and fish oil with BSF meal and corn oil, and their effects on growth, hematology, hematobiochemical, fatty and amino acid composition, and fillet quality.

To the best of the authors’ knowledge, this study is the first study to simultaneously replace fishmeal and fish oil with BSF meal and corn oil, respectively, in diets for hybrid African catfish (*Heteroclarias*).

## 2. Materials and Methods

### 2.1. Animal Ethics Statement

All experimental procedures were approved by the Animal Welfare Committee of the University of Debrecen, Hungary (7/2025/DEMÁB) and complied with the European Union Directive (2010/63/EU) regarding animal experiments.

### 2.2. Experimental Design

The experiment was performed at the Fish Biology Laboratory, Faculty of Agriculture, Food Science and Environmental Management, University of Debrecen, Hungary. The duration was 8 weeks. The hybrid African catfish used in this experiment were bred and raised in the Fish Biology Laboratory, University of Debrecen. The trial was conducted in a recirculating system, circular plastic tanks (350 L), in a completely randomized design. The recirculation system consisted of two main parts: the rearing units and the filtration unit (mechanical and biological filters). Mechanical filtration was performed using sponges, while biological filtration was performed using plastic biofilters followed by UV sterilization.

Water quality parameters were monitored throughout the experiment following APHA [41] standards. Temperature, oxygen, pH, and conductivity were measured in situ, daily using a probe (HACH HQ30d) while the concentration of ammonia (N‐NH_3_^+^), nitrite (N‐NO_2_^−^), and nitrate (N‐NO_3_^−^) were monitored ex situ, weekly using a spectrophotometer (HACH DR3900 spectrophotometer, Hach Company CO, Ames, Iowa, USA). The averages of water quality parameters measured during the experiment are as follows: Temperature 25.5 ± 1.5°C, DO = 5.3 ± 1 mg/L, pH = 7.5 ± 0.30, NH_4_^+^ = 0.38 ± 0.07 mg/L, NH_3_^−^ = 0.2 ± 0.1 mg/L, NO_2_^−^ = 0.03 ± 0.01 mg/L.

### 2.3. Feed Formulation and Preparation

The BSF meal (defatted) used for this experiment was purchased from GRINSECT, Hódmezővásárhely, Hungary. The product label indicates that it contains crude fat 10%, crude protein 55.7% and moisture 4%. The corn oil (VFI GmbH, Wels, Australia) contains energy 3404 kJ/100 g, and fat 92 g/100 g (from which 12.8, 29, and 51 g are saturated, monounsaturated, and polyunsaturated, respectively). Fishmeal (herring, CP = 67%, fat 9%) and fish oil (cod) were obtained from Dobrcz, Poland. All other ingredients were purchased from local manufacturers/producers within Hungary.

Four isonitrogenous diets (400 g kg^−1^ crude protein) and isolipidic (140 g kg^−1^ crude fat) were formulated according to NRC [42]. The four diets were as follows: fishmeal‐based without including BSF larval meal (Control diet), 16.0% (BSF 25), 26.0% (BSF 50), and 36.0% (BSF 75), where fishmeal was replaced at 0%, 25%, 50%, and 75% with BSF larval meal by weight, respectively. Fish oil was completely replaced with corn oil in the test diets. All ingredients were weighed and mixed in a commercial feed mixer (HECHT 2117, Budapest), adding water at 6% (of total weight) to obtain a homogenous dough mixture. Subsequently, the dough was pelletized at 4.5 mm using a pelletizer machine (BORMANN Pro BFP1100, Germany). The pellets were then dried in an air oven (ALPFRIGO CFD 700, UK) at 50°C for 24 h. The pellets were kept in an airtight bag and stored at a room temperature of 24°C until use.

### 2.4. Stocking, Feeding, and Blood Sample Collection

Fish were acclimatized in the experimental system for 2 weeks prior to the start of the experiment, during which they were fed the same high‐quality commercial diet (AQUA Garant, Austria). 180 hybrid African catfish (12 weeks post‐hatching) with an initial body weight of 200 ± 25 g were randomly distributed in the 12 experimental tanks (45 fish per treatment, 15 fish per tank). Feed was administered by hand at a daily ration of 3% of biomass, three times daily, at 8:30, 13:30, and 18:30 h. Sampling was done weekly.

At the end of the experiment, all fish were individually weighed and measured for total length. Three fish from each tank were sacrificed to obtain organo‐somatic indices (i.e., eviscerated, visceral, peritoneal fat, liver, spleen, fillet weights, intestinal length). Three fish were randomly selected from each tank (nine fish per treatment), slightly anesthetized using clove oil (3 mL /100 L), after which blood sample of 1 mL was drawn from the caudal vein into a heparinized vacuum container (BD Vacutainer tube 2 mL) using a single‐use insulin syringe (1 mL) and needle (Sterican 0.5 × 0.4 mm). The blood samples were placed in an ice‐box and immediately sent to the microbiology laboratory for a full blood count.

### 2.5. Blood Biochemistry

Full blood count was done using URIT‐3000Vet Plus Hematology Analyzer (Orvostechnika Ltd, Budapest) following the instructions of the kit manufacturer. A pre‐diluent method was used where 20 µL of blood was pipetted into 1 mL dilution buffer. Readings were taken for red blood cells (RBC), hemoglobin (HGB), hematocrit (HCT), mean corpuscular volume of red blood cell (MCV), mean corpuscular hemoglobin (MCH), MCH concentration (MCHC), platelets (PLT), white blood cells (WBC), granulocytes (GRAN), and lymphocytes (LYM). To obtain the plasma, the remaining blood samples were centrifuged at 3000 g for 15 min to separate the plasma from the cells. The supernatant was carefully poured into an Eppendorf tube and stored at −20°C for blood plasma analysis. Plasma analysis was carried out using an auto‐analyzer (Lab‐Analyse 10261, Orvos Technika Kft, Budapest) following the instructions of the manufacturer. The following parameters were analyzed: Triglyceride, albumin, total protein, globulin, urea, glucose, creatinine, phosphorus, cholesterol, lactate, Alanine aminotransferase (ALT) and Aspartate aminotransferase (AST).

### 2.6. Histological Analysis

Liver samples were preserved in 4% formalin for later analysis. The samples were rinsed with tap water, transferred to 70% ethanol for dehydration, and then embedded in paraffin wax. Cross‐sections 5 mm thick were made in a rotary microtome. Afterwards, the sections were stained with hematoxylin and eosin, sealed with neutral resin adhesive, and then mounted on glass slides for light microscopy reading. The microphotography was taken with a Nikon Coolpix 4300 digital camera coupled to an Olympus microscope (Tokyo, Japan, B941).

### 2.7. Chemical Analysis

Chemical analysis was performed at the Chemistry Laboratory, University of Debrecen, Hungary, to determine the proximate composition, amino acid, and fatty acid profiles of the diets and fish fillets according to AOAC [43] standard methods. Moisture was achieved by drying and weighing (MSZ ISO 1442:2000), and protein was obtained by the Kjeldahl method (MSZ ISO 937:2002). Crude fat by acid–base hydrolysis, extraction and mass measurement (MSZ ISO 1443:2002), Ash by cremation, then mass measurement (MSZ ISO 5984:1992, withdrawn standards), Crude Fiber by acidic, alkaline extraction (Fibretec) (MSZ EN ISO 6865:2001). Chitin content was determined by subtracting acid‐detergent fiber (ADF) from acid‐detergent lignin (ADL) following the method described in Hahn et al. [44]. The ADF and ADL were initially measured gravimetrically according to MSZ EN ISO 13906:2009.

Amino acids were determined by high‐pressure ion exchange chromatography HPLC (MSZ ISO 13903:2005) using an AAA 500 amino acid analyser (INGOS Ltd, Praha, Czech Republic), with post‐column derivation with ninhydrin and photometric detection at 210 and 254 nm. Amino acid standard mixture (INGOS Ltd, Praha, Czech Republic) was applied as a reference. The recovery was higher than 95%. The amino acids were expressed as a percentage of the original sample weight.

The composition of fatty acids was determined using the fatty acid methyl ester method (MSZ ISO 12966‐4:2015). The fat sample (200–300 mg) was dissolved in 6 mL hexane and 4 mL 0.5M NaOH: MeOH. The solution was heated in an oven at 80 ± 1°C for 10 min. After saponification, the sample was diluted with 5–10 mL of distilled water and the unsaponified materials were extracted with 2 mL of hexane. Following the extraction, the solution was acidified with 0.5 mL 6 M H_2_SO_4_ and the saponified fatty acids were extracted with 2 mL hexane. The purified fraction was treated with 2 mL 14% BF_3_:MeOH at 80 ± 1°C for 30 min in an oven. 2 mL saturated NaCl solution was used. The supernatant hexane phase was applied into a GC vial with dry Na‐sulfate and applied to GC‐FID (Varian GC 3800). Supelco 37‐component FAME mix (Sigma–Aldrich) was used as a reference. Measurements were repeated four times with CV% <5%. Calculated results were expressed as a percentage of the fat content.

### 2.8. Production Performance Indices

Growth performance, nutrient utilization, and organo‐somatic indices were calculated as follows:

Mean weight gaing=Final mean weight– Initial mean weight,

Specific growth rateSGR= Inmean final weight– Inmean initial weight/Timedays×100,

Average daily weight gainADG=weight gaing/timedays,

Relative growth rateRGR=Mean final weight of fish/ Mean initial weight of fish×100,

Thermal growth coefficientTGC=1000×final weight g13/−Initial weight g13//Temperature°C×Days,

Feed conversion ratioFCR=Weight of feed fed/Weight gain of fish,

Protein efficiency ratio= Weight gaing/ Crude protein fedg,

Fulton conditioning factorK=W/L3×100 where W is the wet weightg, and  L  is the standard lengthcm,

Daily feed intakeg=Total feed consumed/no. of fish/no. of days,

Hepatosomatic indexHSI=weight of liver/body weight of fish×100,

Spleen-somatic indexSSI=weight of spleen/body weight of fish×100,

Viscerosomatic indexVSI=weight of visceral/body weight of fish×100,

Fillet yield=weight of fillet/body weight of fish×100,

Carcass yield=eviscerated weight of fish/body weight of fish×100,

Relative gut lengthRGL=Length of fish intestinecm/ total body length of fishcm×100,

Survival rate%=initial number of individuals/final number of individuals×100,

Intraperitoneal fat index=weight of perivisceral fat/body weight of fish×100,

Atherogenic index=120160:+4140×C:+C:/sum n-63+ sum n-+ sum MUFA,

Thrombogenic index=C140160180:+C:+C:/0.5× sum MUFA+0.56× sum n-+33× total n-+ sum n-36/n-,

h/H=C1819182620461833205322532263:n–+C:n–+C:n–+C:n–+C:n–+C:n–+C:n–/C140160:+C:. Where h/H= hypocholesterolaemic/hypercholesterolemic fatty acids.

### 2.9. Data Analyses

All statistical analyses were performed using IBM SPSS 29.0 for Windows software. Normality of distribution was tested using Kolmogorov–Smirnov test. Homogeneity of variances between experimental groups was checked using Levene’s test. Since the assumption of homogeneity of variance between the groups was not violated (*p*  > 0.05), the data were subjected to a one‐way analysis of variance (ANOVA). Tukey’s multiple comparison test was used to determine significant differences between treatments (*p*  < 0.05 was considered significant). Polynomial contrast was applied to check the trend response.

The experimental model: Yij = μ + Ti + εij, where Yij is the dependent variable, μ is the overall mean, Ti is the treatment effect (i = BSF 0, BSF 25, BSF 50 or BSF 75), and ɛij is the random residual error.

## 3. Results

### 3.1. Growth Performance, Nutrient Utilization, and Organosomatic Indices

Table 1 presents a summary of the growth performance and nutrient utilization of African catfish reared on varying levels of BSF larval meal.

**Table 1:** Growth performance and nutrient utilization of hybrid African catfish fed varying levels of BSF larval meal‐based diets for 8 weeks.

Parameters	Test diets				*p*‐Values			
	Control	BSF 25	BSF 50	BSF 75	SEM	ANOVA	L	Q
IW (g)	199.91	200.23	200.33	199.83	0.132	0.533	0.925	0.171
FW (g)	490.84^b^	475.41^b^	491.60^b^	408.24^a^	12.524	0.022	0.014	0.075
WG (g)	290.94^b^	275.18^b^	291.27^b^	208.41^a^	12.483	0.022	0.014	0.077
WG (%)	145.52^b^	137.45^a,b^	145.38^b^	104.30^a^	6.211	0.022	0.014	0.080
ADG (g)	5.20^b^	4.91^b^	5.20^b^	3.72^a^	0.223	0.022	0.014	0.077
RGR	2.46^b^	2.37^a,b^	2.45^b^	2.33^a^	0.062	0.022	0.014	0.080
SGR	0.70^b^	0.67^a,b^	0.70^b^	0.55^a^	0.021	0.018	0.011	0.061
TGC	1.43^b^	1.37^a,b^	1.43^b^	1.10^a^	0.049	0.019	0.012	0.070
FI	8.87	8.55	9.26	8.17	0.200	0.278	0.429	0.337
FCR	1.72^a^	1.75^a^	1.78^a^	2.22^b^	0.036	0.002	0.001	0.014
PER	1.41^a,b^	1.41^a,b^	1.44^b^	1.21^a^	0.034	0.035	0.034	0.044
CF(g/cm^3^)	0.85	0.86	0.84	0.80	0.010	0.329	0.138	0.288
SR (%)	100.00	100.00	100.00	100.00	0.000	0.000	0.000	0.000

*Note*: L, Q = Linear and quadratic polynomial contrasts, respectively. Values are means (*n* = 3), pooled standard error of the mean. Values of different superscripts in a row are significantly different (*p*  < 0.05). The absence of a letter indicates no significant difference between treatments.

Abbreviations: ADG, average daily growth; CF, condition factor; FCR, feed conversion ratio, FI, feed intake (gfish^−1^d^−1^); FW, final weight; IW, initial weight; PER, protein efficiency ratio; RGR, relative growth rate; SEM, standard error of the mean; SGR, specific growth rate (% day^−1^); SR, survival rate; TGC, thermal growth coefficient; WG, weight gain.

Fish readily accepted all diets, and survival rate was 100% in all dietary treatments. Physical observation of the whole body of fish from each dietary treatment showed no body deformities or alteration in skin, fins and eyes. Fish fed BSF 50 diet had the highest final mean body weight (491.60 g) and weight gain (291.27 g), which are similar to fish fed BSF 25 and the Control groups, but significantly higher (*p* = 0.022) than fish fed BSF 75 diet. A similar linear effect was observed in specific growth rate, thermal growth coefficient, average daily growth rate, and relative growth rate. The FCR increases linearly (*p* = 0.001) with increasing levels of BSF larval meal inclusion. Fish fed BSF 75 had the highest ratio (2.22), which was significantly higher (*p* = 0.022) than those in the other groups. The protein efficiency ratio (PER) indicates how well dietary protein is utilized for growth. A higher ratio signifies more efficient utilization. The highest PER value was observed in fish fed BSF 50 (1.44), which was similar to the Control and BSF 25, but significantly higher (*p* = 0.035) than fish fed the BSF 75 diet. Condition factor was similar among the groups (*p* = 0.329).

The results of organosomatic indices (Table 2) showed no significant differences (*p*  > 0.05) among the different dietary treatments.

**Table 2:** Organosomatic Indices (%) of hybrid African catfish fed varying levels of BSF larval meal‐based diets for 8 weeks.

Parameters	Test diets				*p*‐Values			
	Control	BSF 25	BSF 50	BSF 75	SEM	ANOVA	L	Q
HSI	1.42	1.42	1.20	1.20	0.052	0.210	0.062	0.973
SSI	0.05	0.04	0.04	0.04	0.002	0.155	0.069	0.156
VSI	8.45	8.00	7.75	7.66	0.680	0.551	0.194	0.637
IPF	4.00	3.21	3.36	3.57	0.179	0.492	0.507	0.208
RGL	101.23	99.76	103.00	85.00	4.016	0.405	0.233	0.327
F Y	41.55	40.02	41.87	38.00	0.660	0.100	0.090	0.281
CY	85.33	81.81	85.50	81.45	0.940	0.276	0.340	0.885

*Note*: L, Q = Linear and quadratic polynomial contrasts, respectively. Values are means (*n* = 3), pooled standard error of the mean. Values of different superscripts in a row are significantly different (*p*  < 0.05). The absence of a letter indicates no significant difference between treatments.

Abbreviations: CY, carcass yield; FY, fillet yield; HSI, hepatosomatic index; IPF, intraperitoneal fat; RGL, relative gut length; SEM, standard error of the mean; SSI, spleen somatic index, VSI, viscerosomatic index.

### 3.2. Proximate, Amino Acids, and Fatty Acids Composition of African Catfish Fillets

The proximate composition, amino acids and fatty acids profiles of African catfish fillets are presented in Tables 3–5, respectively. No significant differences (*p*  > 0.05) were observed in the different dietary groups for dry matter, crude protein, crude fat, saturated fat, and ash content (Table 3).

**Table 3:** Proximate composition (%, dry matter) of hybrid African catfish fillet fed varying levels of BSF larval meal‐based diets for 8 weeks.

Parameters	Test diets				*p*‐Value			
	Ctrl	BSF 25	BSF 50	BSF 75	SEM	ANOVA	L	Q
Dry matter	25.10	24.69	24.82	25.11	0.090	0.274	0.813	0.070
Protein	18.17	18.14	18.09	18.20	0.059	0.938	0.953	0.609
Crude fat	5.41	5.03	5.19	5.39	0.072	0.188	0.883	0.050
Ash	1.22	1.23	1.24	1.23	0.007	0.839	0.559	0.557

*Note:* L, Q = Linear and quadratic polynomial contrasts, respectively. Values are means (*n* = 3), pooled standard deviations of the mean. Values of different superscripts in a row are significantly different (*p*  < 0.05). The absence of a letter indicates no significant difference between treatments.

Abbreviation: SEM, standard error of the mean.

**Table 4:** Amino acid profile (% dry matter) of hybrid African catfish fillet fed varying levels of BSF larval meal‐based diets for 8 weeks.

Parameters	Test die				*p*‐Values			
	Ctrl	BSF 25	BSF 50	BSF 75	SEM	ANOVA	L	Q
Essential AA								
Arginine	0.78	0.76	0.79	0.80	0.009	0.580	0.429	0.458
Histidine	0.49	0.47	0.48	0.50	0.009	0.822	0.811	0.455
Isoleucine	0.76	0.79	0.80	0.77	0.024	0.696	0.790	0.270
Leucine	1.52	1.50	1.54	1.52	0.042	0.937	0.892	0.983
Lysine	2.46	2.30	2.20	2.31	0.134	0.917	0.667	0.626
Methionine	0.41	0.42	0.44	0.41	0.011	0.819	0.866	0.498
Phenylalanine	0.50	0.50	0.48	0.42	0.039	0.916	0.567	0.715
Threonine	0.66	0.68	0.69	0.65	0.029	0.963	0.905	0.636
Valine	0.73	0.73	0.78	0.73	0.025	0.931	0.844	0.702

Non‐essential AA								
Alanine	1.09	1.07	1.08	1.13	0.012	0.395	0.179	0.304
Asparagine	1.76	1.76	1.76	1.76	0.056	0.860	0.475	0.720
Cysteine	0.10	0.11	0.08	0.10	0.006	0.399	0.423	0.961
Glutamine	2.51	2.56	2.39	2.43	0.107	0.959	0.723	0.976
Glycine	1.08	0.94	0.94	1.07	0.036	0.347	0.871	0.087
Proline	1.31	1.30	1.52	1.44	0.125	0.923	0.630	0.906
Serine	0.63	0.63	0.64	0.63	0.014	0.996	0.959	0.857
Tyrosine	0.37	0.38	0.36	0.31	0.031	0.877	0.513	0.663

*Note:* NB: Tryptophan not detected. L and Q = Linear and quadratic polynomial contrasts, respectively. Values are means (*n* = 3), pooled standard error of the mean. Values of different superscripts in a row are significantly different (*p*  < 0.05). The absence of a letter indicates no significant difference between treatments.

Abbreviations: AA, amino acid; SEM, standard error of the mean.

**Table 5:** Fatty acid profile (% total fatty acid) of hybrid African catfish fillet fed varying levels of BSF larval meal‐based diet for 8 weeks.

Parameters	Test diets				*p*‐Values			
	Ctrl	BSF 25	BSF 50	BSF 75	SEM	ANOVA	L	Q
C12:0 (lauric acid)	1.29^a^	4.66^b^	4.74^b^	6.41^c^	0.564	<0.001	0.001	0.001
C14:0 (mystic acid)	3.13^b,c^	3.00^a,b^	2.75^a^	3.35^c^	0.073	0.003	0.279	0.001
C16:0 (palmitic acid)	25.85	23.93	24.94	23.01	0.479	0.168	0.079	1.000
C17:0 (heptadecanoic acid)	1.30	1.14	1.26	1.25	0.046	0.732	0.948	0.458
C18:0 (stearic acid)	9.85	9.26	9.25	9.01	0.157	0.289	0.089	0.579
Total SFAs	42.06	42.53	41.55	43.75	0.441	0.368	0.317	0.343
C16:1n7 (palmitoleic acid)	3.93^b^	2.66^a^	2.24^a^	2.74^a^	0.198	<0.001	0.001	0.001
C18:1n9c (oleic acid)	38.53^b^	32.61^a^	31.30^a^	32.69^a^	0.928	0.002	0.002	0.004
Total MUFAs	42.82^b^	35.52^a^	33.86^a^	34.32^a^	1.204	0.002	0.001	0.011
C18:2n6c (linoleic acid)	7.63^a^	13.77^b^	17.06^c^	15.12^b^	1.076	<0.001	0.001	0.001
C18:3n3 (α‐Linolenic acid)	4.32^b^	3.16^a,b^	2.82^a^	2.16^a^	0.270	0.007	0.001	0.444
C20:4n6 (ARA)	0.26	0.30	0.41	0.26	0.045	0.773	0.520	0.586
C20:5n3 (EPA)	0.71	0.94	0.79	0.61	0.094	0.719	0.632	0.347
C22:6n3 (DHA)	1.47	2.09	2.48	1.97	0.326	0.801	0.573	0.455
Total PUFAs	15.12^a^	21.95^b^	24.60^b^	21.93^b^	1.233	0.011	0.008	0.013
Sum EPA + DHA	2.07	2.61	2.93	2.38	0.390	0.914	0.757	0.556
DHA/EPA	3.29^a^	4.04^a^	5.62^b^	4.24^a,b^	0.290	0.008	0.019	0.013
EPA/ARA	3.48	3.11	1.54	2.50	0.330	0.170	0.118	0.283
Sum n‐3	6.38	5.77	5.75	5.21	0.396	0.830	0.398	0.966
Sum n‐6	8.74^a^	16.18^b^	18.85^b^	16.71^b^	1.185	<0.001	0.001	0.001
n‐3/n‐6	0.73^b^	0.35^a^	0.30^a^	0.31^a^	0.058	<0.001	0.001	0.004
n‐6/n‐3	1.39^a^	2.85^a,b^	3.53^b^	3.32^b^	0.293	0.011	0.004	0.047
PUFA/SFA	0.36^a^	0.52^b^	0.60^b^	0.50^b^	0.029	0.004	0.006	0.003
Atherogenic index	0.69^a^	0.71^a,b^	0.70^a,b^	0.76^b^	0.011	0.034	0.014	0.188
Thrombogenic index	0.85	0.84	0.85	0.86	0.018	0.990	0.886	0.813
h/H ratio	1.83	1.97	1.99	2.01	0.033	0.195	0.063	0.327

*Note:* FA include: C4:0, C6:0, C8:0, C10:0, C11:0, C13:0, C15:0 C17:0, C20:0, C21:0, C22:0, C23:0, C24:0, C14:1, C15:1, C17:1, C16:1n7, C18:1n9t, C20:1n9, C22:1n9, C24:1n9, C18:2n6t, C20:3n3, C20:2n6, C20:3n6. L, Q, and C = Linear and quadratic polynomial contrasts, respectively. Values are means (*n* = 3), pooled standard error of the mean. Values of different superscripts in a row are significantly different (*p*  < 0.05). The absence of a letter indicates no significant difference between treatments.

Abbreviations: AI, atherogenicity index; h/H, hypocholesterolemic/hypercholesterolemic fatty acids; SEM, standard error of the mean; TI, thrombogenicity index.

The amino acid profile of the catfish fillets (Table 4) indicates no significant difference (*p*  > 0.05) among the various dietary treatments. Leucine and lysine are the most abundant essential amino acids (EAA), while alanine, asparagine, glutamine, glycine, and proline are the most abundant nonessential amino acids observed.

Table 5 shows the fatty acid profile of the hybrid African catfish fillets. In contrast to the observed marked differences in the fatty acid content of the Control diet relative to the BSF larval diets, the fatty acid values of the fillets were similar across treatments. The most abundant saturated fatty acids (SFAs) in fillets were lauric acid (C12:0), mystic acid (C14:0), palmitic acid (C16:0), and stearic acid (C18:0). Lauric acid increased significantly (*p* = 0.001) with increasing levels of BSF larval meal. In all, no significant differences (*p* = 0.368) were observed in the total SFA content of catfish fillets among the dietary treatments and no trend response was observed.

Palmitoleic acid (C16:1n7) and oleic acid (C18:1n9) are the main constituents of monounsaturated fatty acids (MUFAs) in fillets of the experimental catfish. The concentration of palmitoleic acid and oleic acid in fish fillets was significantly higher (*p* = 0.001 and 0.002, respectively) in the Control group compared to the BSF groups with linear and quadratic effects. Consequently, fillets of fish fed the Control diet retained significantly higher concentrations (*p* = 0.002) of total MUFA (42.82%) compared to the BSF groups.

PUFAs consist of short‐chain PUFAs (mainly LA C18:2n6 and α‐linolenic acid [ALA] C18:3n3) and long‐chain PUFAs (mainly arachidonic acid C20:4n6, eicosapentaenoic acid C20:4n3 and docosahexaenoic acid C22:6n3). The levels of LA increased linearly and quadratically across the dietary groups, with the peak mean value observed in fish fed BSF 50%, which was significantly different (*p* = 0.001) from the other groups. The ALA in fillets decreased linearly with increasing levels of BSF larval meal. Fish fed on the Control diet had the highest concentration of ALA (4.32%), which was similar to fish fed BSF 25 but significantly higher (*p* = 0.007) than those fed BSF 50 and 75 diets.

In terms of LC‐PUFA, there were no significant differences or trends in ARA, EPA, and DHA among all the dietary treatments. However, the values are generally higher in fish fed BSF diets compared to those that received the Control diet, resulting in a significantly higher total PUFA (*p* = 0.011) in fish fed BSF larval diets than in the Control group, with observed increased linear and quadratic effects.

The DHA/EPA ratio showed a linear and quadratic relationship. The ratios ranged between 3.29 and 5.62. The highest mean value was observed in fillets of fish fed BSF 50 (5.62), which is comparable to BSF 75 (4.24) but significantly higher (*p* = 0.008) than BSF 25 (4.04) and the Control group (3.29). No significant differences were seen in the summation of n‐3 fatty acids (*p* = 0.830). However, fillets of catfish fed BSF larval diets contain lower n‐3 fatty acids compared to the Control group. On the other hand, the sum n‐6 was significantly higher (*p* = 0.001) in fillets of fish fed on BSF diets than in the Control group, with linear and quadratic effects. The study observed linear and quadrilinear responses in n‐3/n‐6 and n‐6/n‐3 ratios. The n‐3/n‐6 ratio was found to be significantly lower (*p* = 0.001) in the fillets of catfish fed BSF larval diets compared to those fed the Control diet.

Conversely, the n‐6/n‐3 ratio was significantly higher (*p* = 0.011) in fish fed BSF larval diets compared to the Control group. The PUFA/SFA ratio ranges from 0.36 to 0.60 and was significantly higher (*p* = 0.004) in the BSF diets than in the Control group. The highest atherogenic index was observed in BSF 75 (0.76), which was similar to BSF 25 and BSF 50 but significantly higher (*p* = 0.034) than in the fish fed the Control diet (0.69). The thrombogenic index and hypocholesterolemic/hypercholesterolemic ratio (h/H) were not statistically significant across the dietary groups, with *p*‐values of 0.990 and 0.195, respectively.

### 3.3. Blood Biochemistry

#### 3.3.1. Hematological Parameters

Data obtained from the full blood count (Table 6) showed no significant differences (*p*  > 0.05) in all parameters across the dietary treatments. However, some patterns are observed: the BSF groups exhibited higher levels of RBC, HGB, and HCT compared to the Control group. Additionally, the BSF groups showed reduced levels of MCH and MCHC across all the treatments relative to the Control. Conversely, PLT count decreased with increasing dietary BSF levels. WBC and GRAN levels were higher in the BSF groups than in the Control. In contrast, lower levels of LYM were observed in fish fed BSF larval meal diets relative to the Control diet.

**Table 6:** Hematological indices of hybrid African catfish fed varying levels of BSF larval meal‐based diets for 8 weeks.

Parameters	Test diets				*p*‐Values			
	Control	BSF 25	BSF 50	BSF 75	SEM	ANOVA	L	Q
RBC (10^12^/L)	2.89	2.90	3.35	3.14	0.107	0.377	0.222	0.606
HGB (g/dL)	11.79	11.80	13.54	12.42	0.331	0.204	0.219	0.384
HCT (%)	35.23	36.20	38.50	37.55	0.749	0.439	0.018	0.530
MCV (fL)	146.04	149.79	145.53	145.41	0.847	0.212	0.413	0.251
MCH (pg)	40.54	40.49	40.35	39.52	0.207	0.270	0.088	0.348
MCHC (g/dL)	27.79	27.09	27.76	27.20	0.256	0.565	0.452	0.932
PLT (10^9^/L)	8.96	6.63	5.85	4.11	0.708	0.103	0.016	0.827
WBC (10^9^/L)	44.53	46.48	54.76	50.48	1.669	0.130	0.076	0.346
GRAN (%)	19.49	20.39	23.60	21.79	0.750	0.236	0.134	0.363
LYM%	66.99	66.34	63.02	64.24	1.208	0.602	0.285	0.773

*Note:* L, Q = Linear and quadratic polynomial contrasts, respectively. Values are means (*n* = 9), pooled standard error of the mean. Values of different superscripts in a row are significantly different (*p*  < 0.05). The absence of a letter indicates no significant difference between treatments.

Abbreviations: GRAN, granulocyte; HCT, hematocrit, HGB, hemoglobin; LYM, lymphocyte; MCH, mean corpuscular hemoglobin; MCHC, mean corpuscular hemoglobin concentration, MCV, mean corpuscular volume of red blood cell; PLT, platelet; RBC, red blood cells; SEM, standard error of the mean; WBC, white blood cells.

#### 3.3.2. Plasma Biochemical Indices

The plasma biochemical indices of hybrid African catfish fed on graded levels of BSF larval meal diets is shown in Table 7. There were no significant differences (*p*  > 0.05) in most plasma indices, except for globulin, albumin–globulin ratio, and cholesterol. Globulin levels were lower in the BSF groups compared to the Control group, which had the highest value (30.20 g/L), similar to BSF 75 but significantly (*p* = 0.015) higher than BSF 25 and BSF 50. The albumin‐globulin ratio was highest in BSF 50 group, which was comparable to BSF 25 but significantly different (*p* = 0.003) from BSF 75 and the Control groups. Cholesterol levels in the BSF groups were lower compared to the Control group, which had the highest level (8.53 mmol/L), similar to BSF 50 but significantly different (*p* = 0.0017) from BSF 25 and BSF 75 groups. Although not statistically significant, a downward trend was observed in total protein, triglycerides, creatinine, lactate, and urea, whilst an upward trend was noticed in AST. Glucose, albumin, and ALT levels remained stable.

**Table 7:** Plasma biochemical indices of hybrid African catfish fed varying levels of BSF larval meal‐based diets for 8 weeks.

Parameters	Test diets				*p*‐Values			
	Ctrl	BSF 25	BSF 50	BSF 75	SEM	ANOVA	L	Q
Total protein (g/l)	45.30	37.74	37.87	40.04	1204	0.080	0.125	0.038
Albumin (g/l)	15.10	13.37	15.18	11.88	0.658	0.236	0.182	0.542
Globulin (g/l)	30.20^b^	24.37^a,b^	22.68^a^	28.16^a,b^	0.981	0.015	0.297	0.002
A/G ratio	0.50^a^	0.55^a,b^	0.67^b^	0.42^a^	0.027	0.003	0.495	0.002
Glucose (mmol/L)	9.41	7.83	8.39	10.19	0.400	0.152	0.397	0.036
Cholesterol (mmol/L)	8.53^b^	6.80^a^	7.48^a,b^	6.87^a^	0.231	0.017	0.021	0.162
Triglyceride (mmol/L)	1.63	1.33	1.52	1.45	0.093	0.726	0.685	0.552
Creatinine (mmol/L)	23.15	19.28	21.78	22.24	0.873	0.465	0.977	0.233
Lactate (mmol/L)	5.91	5.05	5.33	5.57	0.248	0.682	0.747	0.298
Urea (mmol/L)	2.52	2.13	2.45	1.89	0.116	0.197	0.131	0.712
Phosphorus (mmol/L)	5.48	6.08	5.01	5.00	0.169	0.065	0.080	0.326
ALT (U/l)	29.86	31.15	29.30	28.00	0.814	0.605	0.324	0.440
AST (U/l)	50.18	53.11	53.59	54.68	1.024	0.474	0.145	0.661

*Note:* L, Q = Linear and quadratic polynomial contrasts, respectively. Values are means (*n* = 6), pooled standard error of the mean. Values of different superscripts in a row are significantly different (*p*  < 0.05). The absence of a letter indicates no significant difference between treatments.

Abbreviations: A/G, Albumin Globulin, ALT, alanine aminotransferase, AST, aspartate aminotransferase; SEM, standard error of the mean.

### 3.4. Liver Histology

The liver histological appraisal of hybrid African catfish (Figure 1) across all dietary treatments shows normal sinusoidal organization, with no vacuolization or evidence of inflammation. The hepatocyte has a normal structure, uniform appearance, and distribution; the nuclei are regular and not eccentric.

Figure 1Liver histology of hybrid African catfish fed varying levels of BSF larval meal‐based diets for 8 weeks (HE; ×200, Scale bar = 100 µm). A = Control diet, B = BSF 25, C = BSF 50, D = BSF.

**Figure:**
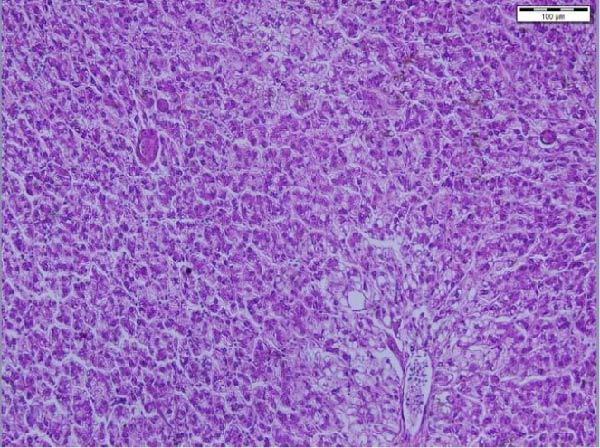
(A)

**Figure:**
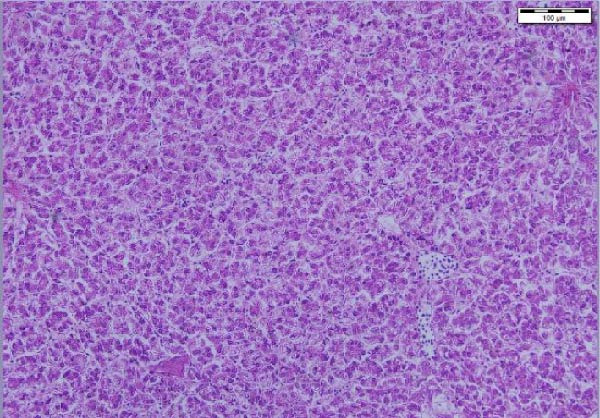
(B)

**Figure:**
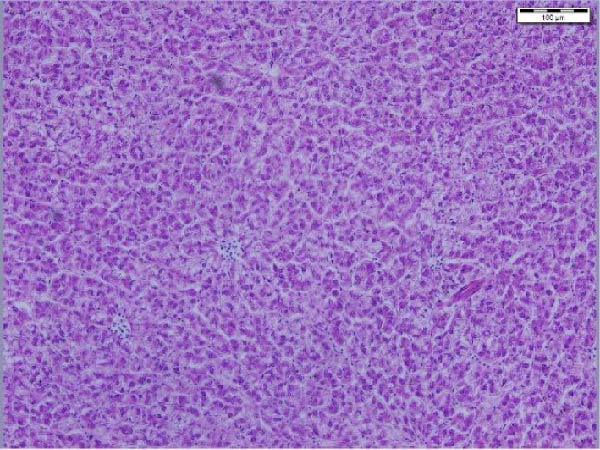
(C)

**Figure:**
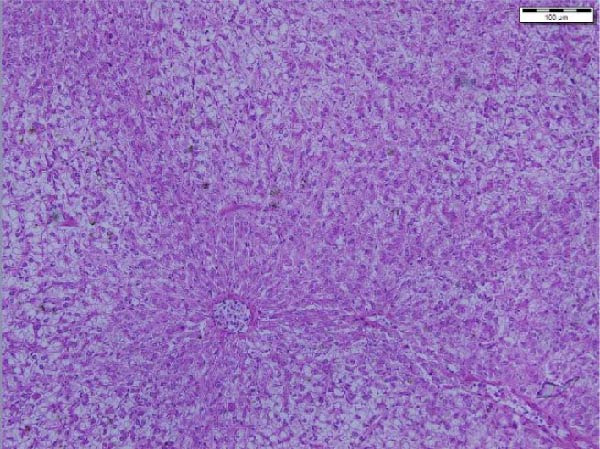
(D)

## 4. Discussion

### 4.1. Diet Composition

Focus has shifted to include not only formulating a diet that satisfies the nutritional requirements of target fish species but also producing fish that meet human dietary requirements. This study demonstrates the feasibility of combined dietary replacement of fishmeal and fish oil with BSF larval meal and corn oil, assessing nutritional safety, physiological parameters, and fillet quality, and offering new insight into the nutritional plasticity of hybrid African catfish, *C. gariepinus* × *H. longifilis*. This approach reflects a more realistic, sustainable, and economically viable strategy for feed formulation.

The experimental diets were formulated to be isonitrogenous, using a conversion factor of N × 6.25. However, because the BSF larval meal contains an appreciable amount of chitin (a non‐protein N) [45, 46], the crude protein content of the diets may be overestimated. Future studies on insect meal should consider using the recommended correctional factor of N × 5.33 [47] or the summation of amino acids.

The amino acid content of BSF larval meal diets is similar to the Control diet (Table 8) and appears to meet the EAA requirements of African catfish [42, 48, 49]. Leucine and lysine were the most abundant EAA observed in all the experimental diets, reflecting their richness in the insect larval meal [4, 50]. The comparable levels of amino acids in all experimental diets suggest that fish were fed a similar quality of protein, and the substitution of fishmeal with BSF larval meal did not negatively affect the amino acid composition of the test diets. This supports previous findings suggesting that the amino acid profile of BSF larvae is well‐balanced and similar to that of fishmeal [16, 51, 52].

**Table 8:** Amino acid composition (%, dry matter) of BSF larval meal‐based diets fed to hybrid African catfish for 8 weeks.

Parameters	Test diets			
	Ctrl	BSF 25	BSF 50	BSF 75
Essential AA				
Arginine	1.79	1.5	1.51	1.41
Histidine	1.26	1.18	1.29	1.16
Isoleucine	1.51	1.51	1.62	1.83
Leucine	2.86	2.79	2.77	2.31
Lysine	3.27	4.41	3.91	3.83
Methionine	1.23	0.89	0.95	1.09
Phenylalanine	1.51	0.91	0.78	0.65
Threonine	2.6	2.02	1.85	1.62
Valine	1.93	1.7	1.71	1.51

Non‐essential AA				
Alanine	2.5	2.63	2.47	2.19
Asparagine	3.98	3.44	3.21	2.39
Cysteine	0.25	2.63	2.47	2.19
Glutamine	6.61	5.49	5.28	4.98
Glycine	2.55	3.05	2.61	2.40
Proline	4.12	4.43	4.94	4.98
Serine	1.79	1.56	1.49	1.30
Tyrosine	0.81	0.64	0.55	0.56

*Note:* NB: Tryptophan not detected.

Abbreviation: AA, amino acid.

The fatty acid composition of corn oil (Table 9) shows that linoleic (C18:2n6) is the most prevalent (55.46%), followed by oleic acid C18:1n9 (30.11%) and palmitic acid C16:0 (10.91%). These results closely align with the previous report by NRC [53].

**Table 9:** Ingredient composition (g/kg, DM) and inclusion levels of BSF larval meal.

Ingredients	Test diets			
	Control	BSF 25	BSF 50	BSF 75
Fishmeal	400	300	200	100
BSF meal	0	160	260	360
JPC 56 soy conc.	200	150	170	160
Blood meal	20	20	20	20
Min./vit. premix	20	20	20	20
Glucose	10	10	10	10
Vitamin C	0.1	0.1	0.1	0.1
Fish oil	91	0	0	0
corn oil	0	85	83	82
Novilpel	10	10	10	10
threonine	8	8	7	8
Lysine	10	11	10	10
Methionine + cystin	5	5	5	5
Tryptophan	1	1	1	1
Wheat	224.9	219.9	203.9	213.9
Total	1000	1000	1000	1000

Proximate composition (%, DM)				
Dry matter	89.96	89.69	89.64	90.22
Crude protein	40.65	40.38	39.81	39.53
Crude fat	13.63	14.02	14.47	15.34
Fiber	1.38	2.29	2.76	3.81
Ash	13.87	11.08	10.86	8.18
Calcium	2.54	2.16	1.97	1.45
Phosphorus	2.18	1.8	1.64	1.14
NFE	20.43	21.92	21.74	23.36
GE (MJ/kg)	13.15	13.74	14.43	15.33
ADF	1.78	2.70	3.28	4.43
Chitin	—	2.46	2.84	3.75

*Note:* NFE = (nitrogen‐free extract) = [100 – (% moisture + protein% + lipid% + ash% + fiber%)].Fishmeal (herring, CP = 67%, fat 9%). BSF larval meal^2^ (defatted) (GRINSECT, Hódmezővásárhely, Hungary), moisture, 4%, crude protein, 55.7%, crude fat, 10%. JPC 56 soy conc. (CP = 56%, crude fat = 3.3%). Blood meal (CP = 95%, crude fat = 1.7%). Corn oil (VFI GmbH, Wels, Australia) Energy, 3404 kJ/100 g; fat 92 g/100 g (SFA, 12.8; MUFA, 29; PUFA, 51 g). Wheat (CP = 12.3%, crude fat 1.3%). Mineral and vitamin premix: Lysine 0.780 %, Methionine 4.257 %, Meth + cistine 4.257%, Calcium, 18.800%; Phosphorus, 9.122%; Sodium, 5.322%; Magnesium, 0.136%; Zinc 4000.000 mg/kg; Copper, 600.000 mg/kg; Iron, 4000.000 mg/kg Manganese, 2668.000 mg/kg; Iodine, 56.000 mg/kg; Selenium, 11.700 mg/kg; Cobalt, 12.000 mg/kg; A vitamin, 440,000.000 IU/kg; D‐3 vitamin, 116,000.000 IU/kg; E vitamin, 2000.000 mg/kg; K‐3 vitamin, 66.000 mg/kg; B‐1 (tiamin) 66.000 mg/kg; B‐2 (riboflavin), 198.000 mg/kg; Pantoth. acid 478.280 mg/kg; B‐6 (piridoxin) 110.000 mg/kg; B‐12(kobalamin) 1.100 mg/kg; Biotine 4.400 mg/kg; 080 B‐3 (nikotinic) 935.770 mg/kg; Folic acid 22.000 mg/kg; Choline‐chloride, 18,000.000 mg/kg; Antioxidants, 55.000 mg/kg; Canthaxanthin 180.000 mg/kg; Phytase, 10,000.000 Ftu/k; Xylanase, 27,000.000 U/kg; Apo‐esther, 92.000 mg/kg; Protease 450.000 U/kg; Sepiolite (E562), 1770.000 mg (Source: KJK‐Agroteam Kft, Dombóvár, Hungary).

Abbreviations: ADF, acid detergent fiber; GE, gross energy.

Palmitic acid is the most abundant SFA in the diets, closely followed by lauric acid. BSF larval meal has been reported to have a high concentration of lauric acid, which contributed to the increased levels of SFA in BSF larval diets compared to the Control diet [16, 54, 55]. Oleic acid (C18:1n9) was the major component of MUFA detected in experimental diets. The concentration of oleic acid decreased in the test diets when fishmeal and fish oil were substituted with BSF larval meal and corn oil, respectively. Consequently, total MUFA decreases across treatments. The present finding aligns with Guerreiro et al. [56] and Abdel‐Tawwab et al. [57] in diets of meagre *Argyrosomus regius* juveniles and European sea bass *Dicentrarchus labrax*, respectively. In contrast, Caimi et al. [58] observed an increasing value of oleic acid in a diet for Siberian sturgeon (*Acipenser baerii*) juveniles when fishmeal was substituted with defatted BSF larvae, resulting in increased MUFA across the groups.

LA and ALA are short‐chain PUFAs that are essential for their role in various physiological processes and for promoting healthy growth and development in fish [59]. In the present experiment, the concentration of LA in the diets was much greater than that of ALA, primarily due to the presence of BSF larval meal and corn oil. LC‐PUFAs, such as ARA, EPA, and DHA, play vital roles in the physiological welfare, growth, and survival of fish [60]. DHA and EPA are regarded as the most important fatty acids for their crucial role in preservation of structural membranes of cells, development of the brain, neural, and visual systems, stress and disease resistance, promote growth and survival of fish, especially during the early stages of development [61–64]. ARA is significant for its role as a precursor to eicosanoid production, which regulates osmoregulation, cardiovascular function, neural control, and reproduction. [65–68]. Most fish are inefficient at synthesizing these LC‐PUFAs; therefore, they obtain these nutrients from their diet [69–71]. While marine ingredients such as fishmeal and fish oil are rich in LC‐PUFA, several researchers have reported that insect meals are either deficient [46, 56, 72, 73] or contain negligible amounts [4, 16, 46] of these important LC‐PUFAs; thus limiting their use in aquafeed and therefore recommend enrichment of insect larvae meant as ingredient for fish feed utilization [39].

The sharp decrease in ARA, EPA, and DHA across the experimental diets could be attributed to a deficiency of these essential fatty acids in BSF larval meal [38, 40, 74] and corn oil (NRC, 1993; [75]). The observed moderate increase in total PUFA across the diets was mainly due to LA, which was by far the most abundant PUFA. Consequently, total n‐3 decreased, and n‐6 increased across the group. Nevertheless, the DHA/EPA ratio (2.3–2.51) meets the nutritional requirements of fish [62]. Similar findings were previously reported by Lock et al. [54], Borgogno et al. [74], Mancini et al. [38], and Hu et al. [76]).

### 4.2. Growth Performance

Replacing fishmeal with up to 75% BSF meal and completely substituting fish oil with corn oil resulted in no mortality, and feed intake was similar (*p*  > 0.05) across all dietary treatments, which positively reflects the fish’s adaptability and acceptability of the new diet. This is consistent with other studies on fishmeal replacement with BSF larval meal [55, 77–79].

Replacing 50% of fishmeal with BSF meal resulted in the highest growth performance (final weight, weight gain, SGR). However, at 75% level, growth performance and nutrient utilization (FCR, PER) significantly decline (*p*  < 0.05). The lower growth in fish fed BSF 75 (360 g kg^−1^ dietary BSF meal), relative to other treatments, was likely not due to palatability or toxicity/pathogenic issues, but rather a deficiency in nutrient uptake and utilization, plausibly due to its high chitin content (3.75%) (Table 9).

Chitin is a polymer of N‐N‐acetylglucosamine, a primary structural component of invertebrate exoskeleton. Although chitinolytic activity was reported in several fish species, including the African catfish [80, 81], most fish lack sufficient chitinolytic enzymes (chitinase and chitobiase) in the gastrointestinal tracts for efficient digestion [72, 82, 83].

Chitin in BSF meal may bind with nutrients, such as proteins and lipids, thereby reducing digestibility and decreasing the bioavailability of EAAs and fatty acids necessary for growth and development ([19, 84, 85]. It has been reported that dietary chitin as low as 1.6% inhibits nutrient uptake in the intestinal tract, resulting in reduced growth in turbot *Scophthalmus maximus* [37]. For example, the apparent digestibility coefficient (ADC) of protein and lipid for BSF larval meal was 63% and 78%, respectively, whereas that of fishmeal was 88%–98% and 98.7% [37]. Villanueva‐Gutiérrez et al. [86] mentioned a chitin content of 2.73% in BSF larval diet as a possible reason for depressed growth in totoaba (*T. macdonaldi*). On the contrary, Piccolo et al. [87] found no significant reduction in protein and lipid digestibility in sea bream *Sparus aurata* fed dietary 500 g kg^−1^ defatted *Tenebrio molitor*, which contains 4.6% chitin.

The bioactive compounds in insect meal (chitin, antimicrobial peptides, lauric acid) may positively impact fish’s immune system and gut health due to their immunostimulant properties [88–90]. However, high inclusion of these compounds may divert energy towards innate immune responses and increase cell turnover, thereby reducing the proportion of dietary energy and protein available for fish growth [91], potentially raising the FCR.

Replacing fishmeal and fish oil with BSF meal and corn oil increases the dietary n‐6/n‐3 ratio, significantly reduces the availability of EPA and DHA, which are essential for maintaining membrane function, promoting growth, and enhancing metabolic efficiency [62, 67]. A deficiency in n‐3 LC‐PUFAs may result in reduced dietary energy available for growth and increased maintenance energy requirements [16, 42, 92].

The observed linear increase in FCR (*p* = 0.001) in a dose‐dependent manner aligns with previous findings by Li et al. [93], Caimi et al. [58], and Mastoraki et al. [94], for Jian carp, Siberian sturgeon and European sea bass, respectively. Similarly, Nayak et al. [95] observed an increasing FCR with increasing substitution of fish oil with linseed oil in diets for silver barb (*Puntius gonionotus*) fingerlings. In contrast, Lock et al. [54] observed a decline in FCR with higher levels of BSF larval meal in diets for Atlantic salmon, possibly due to increased fat deposition in the organs.

The present study supports previous findings by Adeoye et al. [21] and Xiao et al. [96] in which replacement of fishmeal with BSF meal up to 50% (75 g kg^−1^ dietary inclusion) and 48% (223 g kg^−1^) in African catfish (*C. gariepinus*) and yellow catfish (*Petteobagrus fulvidraco*), respectively, did not have adverse effects on fish growth and body composition. Replacing 50% of fishmeal (150 g kg^−1^) with BSF larval meal in the diet for Chinese large‐mouth catfish (*Silurus merdionalis*) for 56 days resulted in significantly improved growth performance compared to the fishmeal‐based control diet [97].

Additionally, a high dietary inclusion of BSF up to 400 g kg^−1^ (50% FM replacement) did not affect the growth and survival rate of rainbow trout *Oncorhynchus mykiss* [55]. Similar observations were made in the Jian carp *Cyprinus carpio var. Jian* [93] and the zebra fish *Danio rerio* [98]. Additionally, in various insect species, fishmeal could be replaced with the yellow mealworm (*Tenebrio molitor*) larval up to 50% (corresponding to 120 and 400 g kg^−1^) meal in diets for the common catfish, *Ameiurus melas* and the blackspot sea bream, *Pagellus bogaraveo*, respectively [99, 100] without impairing growth and nutrient utilization. Substituting fishmeal with housefly maggot *Musca domestica* meal up to 50% (180 g kg^−1^) in diets for Nile tilapia *Oreochromis niloticus* has no significant impact on growth, flesh quality and innate immunity [101].

The present finding contradicts other studies, which indicate that substituting fishmeal with BSF larval meal at levels above 50% does not negatively impact the growth performance of African catfish. Replacing 75% of fishmeal with BSF meal (171.8 g kg^−1^) did not compromise the growth or health of African catfish [20]. Additionally, Olaniyi and Salau [102] reported that incorporating 436.5 g kg^−1^ (75% FM replacement) of housefly maggot meal into the diet did not adversely affect the growth of African catfish. Similarly, including lesser mealworm (*Alphitobius diaperinus*) meal at 157.5 g kg^−1^ (75% FM replacement) in diet for European perch (*Perca fluviatilis*) did not compromise production performance [103].

Complete replacement of fishmeal with BSF larval meal has been reported for the Atlantic salmon [46, 54, 104] and for European sea bass (*Dicentrarchus labrax*; [57]). Kishawy et al. [105] reported that substituting fishmeal with BSF meal up to 100% (100 g kg^−1^) in Nile tilapia diet did not impair growth or feed efficiency. Similarly, Taufek et al. [106] found that complete replacement of fishmeal with cricket (*Gryllus bimaculatus*) meal (300 g kg^−1^) supports healthy growth and nutrient utilization in African catfish. Furthermore, fishmeal was entirely replaced with defatted housefly maggot meal in the diets for African catfish *C. gariepinus* [107, 108] and Nile tilapia *Oreochromis niloticus* [107] without negatively affecting fish growth or health. The differences may be due to factors such as fish species and size, the BSF larval matrix, the processing method of the larval ingredient or the actual dietary dosage of larval meal used in the feed formulation [109, 110].

Previous studies have demonstrated successful partial or total dietary fish oil substitution with vegetable oils and/or animal fat such as BSF larval oil, as was reported in African catfish *C. gariepinus* [111–113], *H. longifilis* [114], Nile tilapia [115, 116], Atlantic salmon [117], and Jian carp [118]. In a 10‐week study on the striped catfish *P. hypophthalmus*, [119] examined BSF larval oil and a series of vegetable oils (moringa, black cumin seed, and flax seed) as a complete replacement for fish oil (23 g kg^−1^). Each diet was formulated to contain 80 g kg^−1^ fishmeal and 174 g kg^−1^ BSF larval meal. The authors concluded that the growth and feed efficiency metrics (final body weight, weight gain, SGR, TGC, and FCR) of fish fed BSF larval oil and moringa oil diets were comparable to those of fish fed the fish oil‐based control diet. Similarly, Babalola et al. [120] reported significant growth and feed efficiency in *H. longifilis* when fed a diet containing 398 g kg^−1^ fishmeal and a complete replacement of 60 g kg^−1^ fish oil with palm kernel oil. A complete replacement of a 1:1 mixture (25 g kg^−1^) of fish oil and rapeseed oil with BSF larval oil in the diet for hybrid African catfish (*C. gariepinus* × *H. longifilis*) did not affect growth and feed utilization [121].

In contrast to the present study, Lin and Shiau [28] found that a complete substitution of fish oil (40 g kg^−1^) with corn oil in a diet for grouper (*Epinephelus malabaricus*) resulted in depressed growth, which was attributed to the low DHA/EPA ratio as a result of the dietary change. However, fish that were fed diets containing a blend of fish oil and corn oil (3:1) and (1:1) exhibited similar high growth rates and nutrient efficiency as those fed the fish oil control diet.

No significant differences were seen in organosomatic indices (HSI, SSI, VSI, IPF, RGL, carcass yield, and fillet yield) in fish fed BSF larval diets compared to the Control group. This suggests that the alternative ingredients did not adversely affect protein and lipid deposition at the organs and carcass levels. These findings align with similar studies in African catfish *C. gariepinus* [20, 22], striped catfish [122], Jian carp *Cyprinus carpio* var. Jian [123], Japanese sea bass *Lateolabrax japanicus* [124] and European sea bass *Dicentrarchus labrax* [125], where substitution of fishmeal with BSF larval meal had no significant influence on HSI, SSI, VSI, and IPF. Similarly, Tran et al. [103] reported that feeding lesser mealworm (*Alphitobius diaperinus*) meal to European perch does not influence the organosomatic indices (HSI, SSI, VSI, MFI, fillet yield).

### 4.3. Proximate Composition, Amino and Fatty Acid Profile, Fillet Nutritional Quality

In this study, the incorporation of BSF larval meal and corn oil in the diets of hybrid African catfish had no significant influence on the proximate composition of the fillets. These findings align with Gebremichael et al. [22], Abdel‐Tawwab et al. [57], and Busti et al. [126], who found no significant differences in dry matter, crude protein, crude fat, and ash content in African catfish *C. gariepinus*, European seabass *Dicentrarchus labrax*, and sea bream *Sparus aurata*, respectively. Similarly, Wang et al. [101] found no significant difference in the proximate composition of Nile tilapia fillets fed diets containing housefly (*Musca domestica*) maggot in dry matter, crude protein, crude lipid, ash, and gross energy. However, some authors observed differences in dry matter, lipid, and ash content in fillets of rainbow trout and European sea bass fed on BSF larval‐based diets [72, 127]. The inclusion of BSF larval meal and corn oil in the test diets did not affect the amino acid profile of the fillets, as the values are similar across all treatments. This contrasts with report by Iaconisi et al. [128], which found significant differences in some amino acids, notably histidine and leucine, in the muscle of gilthead sea bream and rainbow trout fed yellow mealworm (*Tenebrio molitor*) larval‐based diets.

The fatty acid profile of fish fillet, primarily triacyl glycerides (Table 5), generally reflects the fatty acid composition of the diets. However, some fatty acids are either retained in the flesh in the same proportion or selectively utilized [129, 130].

Lauric acid and palmitic acid are the most abundant SFA in BSF larvae meal [16, 39, 57]. The same pattern was seen in the test diets. The notable increase in lauric acid, along with stable levels of palmitic acid and stearic acid, resulted in unchanged total SFA across the groups. Palmitic and stearic acids are long‐chain fatty acids (LCFAs) that are less affected by short‐term dietary changes due to their metabolic stability and slower oxidation rates; therefore, they are readily retained in tissues [131]. In contrast, lauric acid, a medium‐chain fatty acid (MCFA), is rapidly metabolized for energy, resulting in low tissue retention [54, 56]. This was evidenced by the attenuation of lauric acid in fillets relative to diets. Similar observations were made in Jian carp [118] and Atlantic salmon [46, 54]. The significant decrease in sum MUFA in fish that received BSF diets may be largely due to the low oleic acid content found in BSF meal, which was reflected in the fatty acid composition of the diets (Table 10).

**Table 10:** Fatty acid composition (% total fatty acids) of corn oil and experimental diets fed to hybrid African catfish for 8 weeks.

Parameters	Test diets				
	Corn oil	Ctrl	BSF 25	BSF 50	BSF 75
C12:0 (lauric acid)	—	0.37	11.11	13.85	20.59
C14:0 (mystic acid)	—	3.72	3.83	4.01	4.91
C15:0 (pentadecanoic acid)	—	0.58	0.23	0.14	0.08
C16:0 (palmitic acid)	10.91	24.01	18.96	17.11	15.45
C17:0 (heptadecanoic acid)	—	1.48	0.37	0.48	0.25
C18:0 (stearic acid)	1.67	7.68	4.97	4.1	3.06
C20:0	0.35	0.28	0.21	0.20	0.16
C22:0	0.08	0.13	0.10	0.06	0.05
Total SFA	13.02	38.33	40.45	40.37	45.12
C16:1n7 (palmitoleic acid)	0.12	4.23	2.62	2.31	2.32
C17:1 (heptadecanoic acid	—	0.14	0.07	0.05	0.03
C18:1n9 (oleic acid)	30.11	29.77	25.51	24.27	21.99
Total MUFA	30.41	34.14	28.2	26.63	24.34
C18:2n6c (linoleic acid)	55.46	11.76	24.63	28.51	28.15
C18:3n3 (α‐Linolenic acid)	1.11	4.61	2.25	1.61	1.03
C20:4n6 (ARA)	—	0.47	0.19	0.12	0.06
C20:5n3 (EPA)	—	3.25	1.3	0.85	0.37
C22:6n3 (DHA)	—	7.41	2.98	1.91	0.93
Total PUFA	56.57	27.53	31.35	33.00	30.54
Sum EPA + DHA	—	10.66	4.28	2.28	1.30
EPA/ARA	—	6.91	6.84	7.08	6.17
DHA/EPA	—	2.28	2.29	2.25	2.51
Sum n‐3	1.11	15.27	6.53	4.37	2.33
Sum n‐6	55.46	12.26	24.82	28.63	28.21
n‐3/n‐6	0.02	1.25	0.26	0.15	0.08
n‐6/n‐3	49.96	0.80	3.80	6.55	12.11

*Note:* Fatty acids include: C4:0, C6:0, C8:0, C10:0, C11:0, C13:0, C15:0 C17:0, C21:0, C23:0, C24:0, C14:1, C15:1, C17:1, C16:1n7, C18:1n9t, C20:1n9, C22:1n9, C24:1n9, C18:2n6t, C20:3n3, C20:2n6, C20:3n6.

Conversely, the notable increase in sum PUFA in the BSF groups may be attributed to LA, which is abundant in corn oil, (accounting for ~58% of total fatty acids) [23], resulting in the substantial accumulation of linolic acid in the muscle, raising the level of n‐6 fatty acids and contributing to the observed decrease in the n‐3/n‐6 ratio. A decline in n‐3/n‐6 ratio in fillet is a typical outcome when fishmeal and fish oil are replaced with alternative ingredients such as insect meal and vegetable oils [75, 95, 112, 115, 132, 133].

Despite the marked reduction in dietary LC‐PUFA resulting from the inclusion of BSF meal and corn oil, the levels of ARA, EPA, DHA, and the sum n‐3 fatty acids in the fillet remained relatively stable. This contrasts with increased LA and decreased ALA in fillet tissue, parallel with dietary supply. This divergent response does not suggest enhanced de novo biosynthesis from C18 precursors, as there was no dominant compensatory mechanism, leading to either conservation of ALA or increased EPA/DHA deposition in fillet tissue [42, 134–136]. Instead, it likely reflects preferential retention or selective conservation of LC‐PUFA in muscle tissue, a phenomenon previously documented in various freshwater and euryhaline teleosts such as Atlantic salmon [117, 137], gilthead seabream, European seabass [138], rainbow trout [139, 140], largemouth bass *Micropterus salmoides* [141], and striped catfish *Pangasianodon hypophthalmus* [142], African catfish, *C. gariepinus* [143] when fish oil is partially or totally replaced with an alternative lipid source such as vegetable oils or animal fat.

This retention highlights the physiological importance of these fatty acids (in maintaining cell membrane integrity and synthesizing eicosanoids), resulting in reduced catabolism compared with MUFAs and SFA [10, 11, 65, 131, 144, 145]. Besides, regulation of lipid metabolism in the muscle tissue is species‐specific [10, 11, 146]. It appears that hybrid African catfish specifically modulate LC‐PUFA by selectively oxidizing excess n‐6 and MUFA for β‐oxidation, while sparing EPA and DHA, thus prioritizing their presence in phospholipids, which are essential for structural functions [75, 147, 148].

African catfish are considered relatively lean‐fleshed (< 2%) or low‐fat (2%–4% ww fat) teleosts [149], typically with low levels of intramuscular lipid deposition [150]. The lipids found in their muscle are predominantly structural and are tightly regulated to maintain membrane integrity [10, 11]. Consequently, a short dietary change may have a limited influence on the muscle compared to its impact on the liver and visceral tissues.

From a fish health perspective, a moderate increase in muscle LA (n‐6) and lauric acid (C12:0) may not be inherently detrimental to fish. LA may serve as an efficient energy substrate, while lauric acid is known for its antimicrobial and immunomodulatory properties, which may improve gut health and enhance immune responses [88, 90].

Producing fish that meet human dietary requirements for n‐3/n‐6 fatty acids is essential, as these fatty acids offer numerous health benefits, including the prevention of coronary heart diseases and the reduction of inflammatory disorders [150–152]. This study found that the n‐3/n‐6 ratios in the fillets of fish fed BSF diets (0.30–0.35) were substantially lower than in the Control diet (0.73). Additionally, these ratios were lower than those reported for the muscle of other fish species fed BSF larval‐based diets, such as hybrid African catfish (heteroclarias) [121], African catfish (*C. gariepinus*) [22] and Jian carp (*Cyprinus carpio var. Jian* [118]). The low n‐3 muscle fatty acid profile may reflect a trade‐off between sustainability and optimal lipid nutrition. Nevertheless, the n‐3/n‐6 ratios observed in this study fall within the recommended range (0.25 to 1) for human dietary intake [153].

From a human nutrition perspective, in addition to the n‐3/n‐6 ratio, the nutritional quality of fatty acids in fish muscle could be assessed using various indices, including the PUFA/SFA ratio, the atherogenicity index (AI), the thrombogenicity index (TI) and the hypocholesterolemic/hypercholesterolemic index (h/H) [151]. The significantly improved PUFA/SFA ratio reflects a higher intake of dietary PUFA, particularly LA from corn oil. PUFA is recognized for its cardiovascular benefits in humans, as it helps lower low‐density lipoprotein (LDL) cholesterol levels [154, 155]. In this study, the PUFA/SFA ratio ranged from 0.50 to 0.60, surpassing the minimum threshold of > 0.45 for a healthy diet [156, 157].

The significantly higher AI in fish fed BSF 75 (0.76) relative to those fed the Control diet (0.69), suggests a theoretical potential towards promoting atherosclerosis due to higher levels of SFA. The absence of significant differences in TI across treatments suggests that hybrid African catfish can preferentially retain anti‐thrombotic fatty acids (MUFA and LC‐PUFA) in their muscle tissue despite dietary changes. For fish meat to be considered beneficial for human health in reducing the risk of coronary heart disease, both AI and TI levels should be below 1.0 [55]. In the current study, both AI and TI levels were below 1.0 and within the range (0.48–0.83) reported for various fish species [156]. The stable h/H index further confirms that dietary changes did not compromise the cholesterol‐lowering potential of catfish fillets.

Overall, the nutritional quality indices suggest that the fillets of hybrid catfish raised on BSF‐corn oil‐based diets are nutritionally acceptable. Increasing consumers’ awareness and acceptance of fish cultured on insect‐based diets could enhance human nutrition and contribute to sustainability by reducing reliance on finite marine resources while supporting circular bioeconomy principles.

### 4.4. Blood Biochemistry and Liver Histology

#### 4.4.1. Hematological Parameters

Blood biochemical parameters, such as full blood count and plasma or serum analysis, are utilized to evaluate the physiological and health status of fish. They offer comprehensive insights into oxygen transport capacity, immune potential, nutritional status, stress level, disease, and toxicity in fish [32, 158–160]. Hematological parameters of fish are influenced by internal factors such as species, age, sex, and inflammation, while external factors include nutrition, stocking density, handling, and water quality parameters [158, 161].

The hematological parameter values recorded herein are within the recommended range for *C. gariepinus* [162, 163] and closely align with those reported by other researchers [159, 164]. The hematological profile showed no statistically significant differences across the various treatments, indicating that incorporating BSF meal and corn oil into the diets for African catfish did not negatively impact fish blood health or compromise the physiological condition. This finding is consistent with previous studies by Fawole et al. [20] and Adeoye et al. [21], which showed that dietary inclusion of BSF larval meal at 171.8 and 150 g kg^−1^, respectively, did not significantly alter the hematological profile of the African catfish. Nevertheless, some discernible patterns were identified, which may be viewed as indicative rather than definitive.

The moderately increased levels of RBC, HGB, and HCT alongside stable MCV but mildly reduced MCH and MCHC in the BSF group relative to the Control group may suggest stimulation of erythropoiesis with similar cell size but slightly less HGB‐rich content (hypochromia), which may potentially reflect a suboptimal supply of essential hematopoietic micronutrients such as iron, vitamin B12, and folate. Chitin in BSF meal may bind with functional iron, thereby reducing its bioavailability for heme synthesis [158, 165, 166]. Additionally, replacing fish oil with corn oil (high in LA but negligible EPA/DHA) may alter cell membrane composition, potentially increasing lipid peroxidation, thereby impairing heme synthesis, despite an increase in total RBC production [165, 167]. The increased erythropoietic output could be a compensatory response to sustain adequate oxygen transport under the new dietary regime [167, 168].

The moderate rise in leukocyte count (WBC) and granulocytes, alongside a subtle reduction in LYM count, in the BSF diets compared to the fishmeal control diet, may reflect a mild activation of the immune response and a shift in the leukocyte profile towards neutrophils or monocytes [160, 169]. This may suggest a dominance of the innate immune response over adaptive immunity, possibly triggered by bioactive compounds in BSF meal (e.g., chitin, AMPs, and lauric acids) and corn oil lipid milieu (which favors arachidonic acid‐derived eicosanoids and granulocyte recruitment) [79, 83, 170]. These factors tend to stimulate innate immunity, leading to increases in neutrophils, monocytes, and total WBC count, resulting in a relative decrease in LYMs [159]. A moderate reduction in PLT count may result from minor modulation of thrombopoiesis influenced by dietary factors such as chitin and fatty acids, which may affect blood clotting parameters and PLT regulation [33, 171, 172]. Overall, the hematological patterns suggest that incorporating BSF meal and corn oil in hybrid African catfish diets does not cause overt hematological impairments, although there may be mild immune‐modulating effects.

#### 4.4.2. Plasma Biochemical Indices

The results of plasma biochemical indices indicate that replacing fishmeal and fish oil with BSF larval meal and corn oil in the diet did not adversely affect the African catfish, as most parameters remained stable. However, significant decreases in globulin and cholesterol levels were observed. The reduction likely reflects physiological and dietary adaptation rather than impairment of immune or metabolic function. In teleosts, plasma globulin encompasses immunoglobulins and acute‐phase proteins [158, 160, 173]. The observed reduction, in the absence of mortality, elevated liver enzymes (AST and ALT) and inflammatory histology, while total protein and hematological indices remained unaffected, suggests a mild shift towards activation of innate immune function rather than immunosuppression [158].

Similarly, the reduction in plasma cholesterol levels, which may be a positive health indicator, may be attributed to the dietary plant‐based corn oils and BSF meal components, such as phytosterols and chitin, which are associated with hypocholesterolemic effects [27, 93, 114, 174]. Additionally, the reduction in cholesterol level may be attributed to lauric and other MCFAs, which are rapidly oxidized for energy, reducing the fat available for storage as adipose tissue, and in turn limit liver lipid accumulation [54, 93, 174].

Furthermore, the normal levels of triglyceride and hepatosomatic indices across the treatments suggest lipid digestion and metabolic regulation were not adversely affected [175, 176]. The finding aligns with previous reports on African catfish *H. longifilis* [114], European sea bass [14], and Japanese sea bass [124] fed BSF larval diets. In contrast, Egessa et al. [121], Fawole et al. [20], Sudha et al. [119], and Tran et al. [92] reported that hybrid African catfish, African catfish, striped catfish and European perch (*Perca fluviatilis*) maintained stable serum cholesterol fed BSF‐based diets.

Other indices, including albumin, glucose, lactate, creatinine, and urea, showed no significant differences, suggesting that the dietary modifications did not adversely affect energy metabolism, muscle function, renal health, or nitrogen excretion [159, 175, 177, 178]. Overall, the biochemical responses suggest that the hybrid African catfish can maintain metabolic and immune homeostasis despite the dietary changes.

#### 4.4.3. Liver Histology

The liver is the primary site of manifestation for dietary imbalances due to its central role in metabolism, making it susceptible to cellular injury, lipid accumulation (fatty liver), or inflammatory responses [164]. Histological examination of the liver of the African catfish (Figure 1) indicates that the dietary modification did not adversely affect hepatic structure or function. The normal hepatocyte structure, regular nuclear shape, normal sinusoidal organization, and no obvious vacuolization, inflammation, or necrosis indicate that both nutrient metabolism and detoxification processes were maintained. The finding is further supported by the hematological and plasma biochemistry results, which show no significant differences across most parameters. Contrarily, Jimoh et al. [179] noticed vacuolation of the hepatocytes of the liver in *C. gariepinus* fed cooked sesame meal‐based diets.

## 5. Conclusion

The study evaluated the effects of replacing fishmeal and fish oil with BSF and corn oil on growth performance, amino acids, fatty acids composition, blood biochemistry, and liver histology of hybrid African catfish. Fish readily accepted all diets, and no mortality was recorded. The amino acid content of fillets of fish fed the BSF diets was relatively similar to that of the fishmeal‐based Control treatment. The ARA, EPA DHA, and sum n‐3 PUFA contents in fillets of fish fed BSF larval diets were comparable to the Control group. Results of the blood biochemistry and liver histology indicate that the dietary changes did not compromise the general health status of cultured African catfish. Overall, the findings of this study suggest that BSF larval meal may partially replace dietary fishmeal with up to 50% (200 g kg^−1^) and corn oil may completely replace fish oil, in a practical diet for hybrid African catfish without exerting adverse impacts on growth, feed conversion, fillet nutritional quality, and physiological well‐being.

**Nomenclature**
ADC: Apparent digestibility coefficient
ALA: Alpha linolenic acid
AMP: Anti‐microbial peptides
AI: Atherogenic index
ARA: Arachidonic acid
BSF: Black soldier fly
DHA: Docosahexaenoic acid
DM: Dry matter
EAA: Essential amino acids
EPA: Eicosapentaenoic acid
FA: Fatty acids
FAO: Food and Agriculture Organization
FCR: Feed conversion ratio
h/H: Hypocholesterolemic/hypercholesterolemic index
HSI: Hepatosomatic index
LA: Linoleic acid
LC: Long‐chain
MCFA: Medium‐chain fatty acids
MUFA: Monounsaturated fatty acids
n‐3: Omega‐3 fatty acids
n‐6: Omega‐6 fatty acids
NEAA: Non‐essential amino acids
NRC: National Research Council
PUFA: Polyunsaturated fatty acids
SC: Short chain
SFA: Saturated fatty acids
SGR: Specific growth rate
SSI: Spleen‐somatic index
TI: Thrombogenic index
VSI: Viscerosomatic index.

## Author Contributions

**Christopher Teye-Gaga:** conceptualization, data curation, formal analysis, methodology, writing – original draft, writing – review and editing. **Péter István Molnár:** data curation, supervision, writing – review and editing. **Attila Kertész:** data curation, supervision, writing – review andediting. **John Kiguru Maina:** data curation, supervision, writing – review and editing. **Elshafia Ali Hamid Mohammed:** data curation, supervision, writing – review and editing. **Gabriella Gulyás:** data curation, supervision, validation, writing – review and editing. **Levente Czeglédi:** data curation, project administrator, supervision, validation, writing – review and editing. **Milán Fehér:** data curation, project administrator, supervision, validation, writing – review and editing. **Péter Bársony:** conceptualization, data curation, methodology, supervision, validation, writing – review and editing.

## Funding

No funding was received for this manuscript.

## Conflicts of Interest

The authors declare no conflicts of interest.

## Data Availability

All data generated or analyzed during this study are included in this published article.

## References

[1] FAO , The State of World Fisheries and Aquaculture 2024. Blue Transformation in Action, 2024, FAO, 10.4060/cd0683en.

[2] Maulu S. , Langi S. , and Hasimuna O. J. , et al.Recent Advances in Utilization of Insects as an Ingredient in Aquafeeds: A Review, Animal Nutrition. (2022) 11, 334–349, 10.1016/j.aninu.2022.07.013. 36329686 PMC9618972

[3] van Huis A. , Potential of Insects as Food and Feed in Assuring Food Security, Annual Review of Entomology. (2013) 58, no. 1, 563–583, 10.1146/annurev-ento-120811-153704, 2-s2.0-84873860862. 23020616

[4] Makkar H. P. S. , Tran G. , Heuzé V. , and Ankers P. , State-of-the-Art on the Use of Insects as Animal Feed, Animal Feed Science and Technology. (2014) 197, 1–33, 10.1016/j.anifeedsci.2014.07.008, 2-s2.0-85027932786.

[5] van Huis A. , Edible Insects Contributing to Food Security?, Agriculture & Food Security. (2015) 4, no. 1, 10.1186/s40066-015-0041-5, 2-s2.0-85005982523, 20.

[6] Rumpold B. A. and Schlüter O. K. , Nutritional Composition and Safety Aspects of Edible Insects, Molecular Nutrition & Food Research. (2013) 57, no. 5, 802–823, 10.1002/mnfr.201200735, 2-s2.0-84876440123. 23471778

[7] Kenis M. , Koné N. , and Chrysostome C. A. A. M. , et al.Insects Used for Animal Feed in West Africa, Entomologia. (2014) 2, no. 2, 107–114, 10.4081/entomologia.2014.218.

[8] Ferrer Llagostera P. , Kallas Z. , Reig L. , and Amores de Gea D. , The use of Insect Meal as a Sustainable Feeding Alternative in Aquaculture: Current Situation, Spanish Consumers’ Perceptions and Willingness to Pay, Journal of Cleaner Production. (2019) 229, 10–21, 10.1016/j.jclepro.2019.05.012, 2-s2.0-85065503084.

[9] Meijer N. , Safitri R. A. , Tao W. , and Hoek-Van den Hil E. F. , Review: European Union Legislation and Regulatory Framework for Edible Insect Production – Safety Issues, Animal. (2025) 19, 10.1016/j.animal.2025.101468, 101468. 40133170

[10] Xu H. , Turchini G. M. , and Francis D. S. , et al.Are Fish What They Eat? A Fatty Acid’s Perspective, Progress in Lipid Research. (2020) 80, 10.1016/j.plipres.2020.101064, 101064. 33010278

[11] Xu X. , Ji H. , Belghit I. , and Sun J. , Black Soldier Fly Larvae as a Better Lipid Source Than Yellow Mealworm or Silkworm Oils for Juvenile Mirror Carp (*Cyprinus carpio* var. Specularis), Aquaculture. (2020) 527, 10.1016/j.aquaculture.2020.735453, 735453.

[12] Newton L. , Sheppard C. , Watson D. W. , Burtle G. , and Dove R. , Using the Black Soldier Fly, Hermetia illucens, as a Value-Added Tool for the Management of Swine Manure, 2005, Waste Management Programs. North Carolina State University, http://www.cals.ncsu.edu/waste_mgt/smithfield_projects/phase2report05/cd,web%20files/A2.p.

[13] Bußler S. , Rumpold B. A. , Jander E. , Rawel H. M. , and Schlüter O. K. , Recovery and Techno-Functionality of Flours and Proteins From Two Edible Insect Species: Mealworm (*Tenebrio molitor*) and Black Soldier Fly (*Hermetia illucens*) Larvae, Heliyon. (2016) 2, no. 12, 10.1016/j.heliyon.2016.e00218, 2-s2.0-85007494826, e00218. 28054035 PMC5198854

[14] Magalhães R. , Sánchez-López A. , Leal R. S. , Martínez-Llorens S. , Oliva-Teles A. , and Peres H. , Black Soldier Fly (*Hermetia illucens*) Pre-Pupae Meal as a Fish Meal Replacement in Diets for European Seabass (*Dicentrarchus labrax*), Aquaculture. (2017) 476, 79–85, 10.1016/j.aquaculture.2017.04.021, 2-s2.0-85018579300.

[15] Schiavone A. , De Marco M. , and Martínez S. , et al.Nutritional Value of a Partially Defatted and a Highly Defatted Black Soldier Fly Larvae (*Hermetia illucens* L.) Meal for Broiler Chickens: Apparent Nutrient Digestibility, Apparent Metabolizable Energy and Apparent Ileal Amino Acid Digestibility, Journal of Animal Science and Biotechnology. (2017) 8, no. 1, 1–9, 10.1186/s40104-017-0181-5, 2-s2.0-85020426562. 28603614 PMC5465574

[16] Henry M. , Gasco L. , Piccolo G. , and Fountoulaki E. , Review on the Use of Insects in the Diet of Farmed Fish: Past and Future, Animal Feed Science and Technology. (2015) 203, no. 1, 1–22, 10.1016/j.anifeedsci.2015.03.001, 2-s2.0-84928339785.

[17] Belghit I. , Liland N. S. , and Waagbø R. , et al.Potential of Insect-Based Diets for Atlantic Salmon (*Salmo salar*), Aquaculture. (2018) 491, 72–81, 10.1016/j.aquaculture.2018.03.016, 2-s2.0-85051825922.

[18] Barroso F. G. , de Haro C. , Sánchez-Muros M.-J. , Venegas E. , Martínez-Sánchez A. , and Pérez-Bañón C. , The Potential of Various Insect Species for Use as Food for Fish, Aquaculture. (2014) 422-423, 193–201, 10.1016/j.aquaculture.2013.12.024, 2-s2.0-84891954632.

[19] Agbohessou P. S. , Mandiki S. N. M. , and Gougbédji A. , et al.Total Replacement of Fishmeal by Enriched-Fatty Acid *Hermetia illucens* Meal Did Not Substantially Affect Growth Parameters or Innate Immune Status and Improved Whole Body Biochemical Quality of Nile Tilapia Juveniles, Aquaculture Nutrition. (2021) 27, no. 3, 880–896, 10.1111/anu.13232.

[20] Fawole F. J. , Adeoye A. A. , Tiamiyu L. O. , Ajala K. I. , Obadara S. O. , and Ganiyu I. O. , Substituting Fishmeal With *Hermetia illucens* in the Diets of African Catfish (*Clarias gariepinus*): Effects on Growth, Nutrient Utilization, Haemato-Physiological Response, and Oxidative Stress Biomarker, Aquaculture. (2020) 518, 10.1016/j.aquaculture.2019.734849, 734849.

[21] Adeoye A. A. , Akegbejo-Samsons Y. , Fawole F. J. , and Davies S. J. , Preliminary Assessment of Black Soldier Fly (*Hermetia illucens*) Larval Meal in the Diet of African Catfish (*Clarias gariepinus*): Impact on Growth, Body Index, and Haematological Parameters, Journal of the World Aquaculture Society. (2020) 51, no. 4, 1024–1033, 10.1111/jwas.12691.

[22] Gebremichael A. , Szabó A. , Sándor Z. J. , Nagy Z. , Ali O. , and Kucska B. , Chemical and Physical Properties of African Catfish (*Clarias gariepinus*) Fillet Following Prolonged Feeding With Insect Meal-Based Diets, Aquaculture Nutrition. (2023) 2023, 10.1155/2023/6080387, 6080387. 37674976 PMC10480016

[23] Maina J. G. , Beames R. M. , Higgs D. , Mbugua P. N. , Iwama G. , and Kisia S. M. , Partial Replacement of Fishmeal With Sunflower Cake and Corn Oil in Diets for Tilapia *Oreochromis niloticus* (Linn): Effect on Whole Body Fatty Acids, Aquaculture Research. (2003) 34, no. 8, 601–608, 10.1046/j.1365-2109.2003.00848.x, 2-s2.0-0037969030.

[24] Corn Refiners Association (CRA) , Corn Oil, 2006, 5th edition, CornRefiners Association.

[25] Apraku A. , Huang X. , Ayisi C. L. , and Yusuf A. , Potential of Corn Oil as Alternative Dietary Lipid Source in Aquaculture Feeds, Journal of Applied Sciences. (2019) 19, no. 3, 156–165, 10.3923/jas.2019.156.165.

[26] Dupont J. , White P. J. , and Carpenter M. P. , et al.Food Uses and Health Effects of Corn Oil, Journal of the American College of Nutrition. (1990) 9, no. 5, 438–470, 10.1080/07315724.1990.10720403, 2-s2.0-0025637253. 2258533

[27] Ostlund R. E.Jr, Racette S. B. , Okeke A. , and Stenson W. F. , Phytosterols That Are Naturally Present in Commercial Corn Oil Significantly Reduce Cholesterol Absorption in Humans, American Journal of Clinical Nutrition. (2002) 75, no. 6, 1000–1004, 10.1093/ajcn/75.6.1000. 12036805

[28] Lin Y. H. and Shiau S. Y. , Effects of Dietary Blend of Fish Oil With Corn Oil on Growth and Non-Specific Immune Responses of Grouper *Epinephelus malabaricus* , Aquaculture Nutrition. (2007) 13, no. 2, 137–144, 10.1111/j.1365-2095.2007.00458.x, 2-s2.0-33847339763.

[29] Zhou J. C. , Han D. , Jin J. Y. , Xie S. Q. , Yang Y. X. , and Zhu X. M. , Compared to Fish Meal Oil Alone, A Corn and Fish Oil Mixture Decreases the Lipid Requirement of a Freshwater Fish Species, *Carassius auratus* Gibelio, Aquaculture. (2014) 428-429, 272–279, 10.1016/j.aquaculture.2014.03.029, 2-s2.0-84897948964.

[30] Sáez-Royuela M. , García T. , Carral J. M. , and Celada J. D. , Fish Oil Replacement by a Blend of Vegetable Oils in Diets for Juvenile Tench (*Tinca tinca* Linnaeus, 1758): Effects on Growth Performance and Whole-Body Composition, Animals. (2022) 12, no. 9, 10.3390/ani12091113, 1113. 35565539 PMC9105335

[31] Adewuni A. A. and Olaleye F. V. , Catfish Culture in Nigeria: Progress, Prospects and Problems, African Journal of Agricultural Research. (2011) 6, no. 6, 1281–1285.

[32] Taufek N. M. , Aspani F. , Muin H. , Raji A. A. , Razak S. A. , and Alias Z. , The Effect of Dietary Cricket Meal (*Gryllus bimaculatus*) on Growth Performance, Antioxidant Enzyme Activities, and Haematological Response of African Catfish (*Clarias gariepinus*), Fish Physiology and Biochemistry. (2016) 42, no. 4, 1143–1155, 10.1007/s10695-016-0204-8, 2-s2.0-84958778173. 26886132

[33] Raji A. A. , Alaba P. A. , and Yusuf H. , et al.Fishmeal Replacement With *Spirulina platensis* and *Chlorella vulgaris* in African Catfish (*Clarias gariepinus*) Diet: Effect on Antioxidant Enzyme Activities and Haematological Parameters, Research in Veterinary Science. (2018) 119, 67–75, 10.1016/j.rvsc.2018.05.013, 2-s2.0-85047826025. 29864632

[34] FAO , The State of World Fisheries and Aquaculture 2022. Toward Blue Transformation, 2022, FAO.

[35] Olaleye V. F. , A Review of Reproduction and Gamete Management in the African Catfish *Clarias Gariepinus* (Burchell), Ife Journal of Science. (2005) 7, no. 1, 63–70, 10.4314/ijs.v7i1.32158.

[36] FAO , *Clarias gariepinus* (Burchell, 1822), Cultured Aquatic Species Information Programme. (2019) 3, no. 47, 13.

[37] Kroeckel S. , Harjes A. , and Roth I. , et al.When a Turbot Catches a Fly: Evaluation of A Pre-Pupae Meal of the Black Soldier Fly (*Hermetia illucens*) as Fish Meal Substitute—Growth Performance and Chitin Degradation in Juvenile Turbot (*Psetta maxima*), Aquaculture. (2012) 364-365, 345–352, 10.1016/j.aquaculture.2012.08.041, 2-s2.0-84866725176.

[38] Mancini S. , Medina I. , Iaconisi V. , Gai F. , Basto A. , and Parisi G. , Impact of Black Soldier Fly Larvae Meal on the Chemical and Nutritional Characteristics of Rainbow Trout Fillets, Animal. (2018) 12, no. 8, 1672–1681, 10.1017/S1751731117003421, 2-s2.0-85042233946. 29282157

[39] Alfiko Y. , Xie D. , Astuti R. T. , Wong J. , and Wang L. , Insects as a Feed Ingredient for Fish Culture: Status and Trends, Aquaculture and Fisheries. (2022) 7, no. 2, 166–178, 10.1016/j.aaf.2021.10.004.

[40] Fabrikov D. , Barroso F. G. , and Sánchez-Muros M. J. , et al.Effect of Feeding With Insect Meal Diet on the Fatty Acid Compositions of Sea Bream (*Sparus aurata*), Tench (*Tinca tinca*) and Rainbow Trout (*Oncorhynchus mykiss*) Fillets, Aquaculture. (2021) 545, 10.1016/j.aquaculture.2021.737170, 737170.

[41] APHA , Standard Methods for the Examination of Water and Wastewater, 1999, 20th edition, American Public Health Association.

[42] NRC (National Research Council) , Nutrient Requirements of Fish and Shrimp, 2011, National Academies Press, 327–347.

[43] AOAC , Official Methods of Analysis of the Association of Official Analytical Chemists, 2016, 20th edition, AOAC Inc.

[44] Hahn T. , Roth A. , and Febel E. , et al.New Methods for High- Accuracy Insect Chitin Measurement, Journal of the Science of Food and Agriculture. (2018) 98, 5069–5073, 10.1002/jsfa.9044, 2-s2.0-85052528719. 29604075

[45] Janssen R. H. , Vincken J.-P. , van den Broek L. A. M. , Fogliano V. , and Lakemond C. M. M. , Nitrogen-to-Protein Conversion Factors for Three Edible Insects: *Tenebrio molitor*, *Alphitobius diaperinus*, and *Hermetia illucens* , Journal of Agricultural and Food Chemistry. (2017) 65, no. 11, 2275–2278, 10.1021/acs.jafc.7b00471, 2-s2.0-85016139196. 28252948 PMC5364430

[46] Belghit I. , Liland N. S. , and Gjesdal P. , et al.Black Soldier Fly Larvae Meal Can Replace Fish Meal in Diets of Sea-Water Phase Atlantic Salmon (*Salmo salar*), Aquaculture. (2019) 503, 609–619, 10.1016/j.aquaculture.2018.12.032, 2-s2.0-85059624979.

[47] Boulos S. , Tännler A. , and Nyström L. , Nitrogen-to-Protein Conversion Factors for Edible Insects on the Swiss Market: *T. molitor*, *A. domesticus*, and *L. migratoria* , Frontiers in Nutrition. (2020) 7, 10.3389/fnut.2020.00089, 9. 32754611 PMC7366252

[48] Parker J. , Aquaculture Science, 2012, 3rd edition, Cengage Learning.

[49] Nunes A. J. P. , Sá M. V. C. , Browdy C. L. , and Vázquez-Anon M. , Practical Supplementation of Shrimp and Fish Feeds With Crystalline Amino Acids, Aquaculture. (2014) 431, 20–27.

[50] De Marco M. , Martínez S. , and Hernandez F. , et al.Nutritional Value of Two Insect Larval Meals (*Tenebrio molitor* and *Hermetia illucens*) for Broiler Chickens: Apparent Nutrient Digestibility, Apparent Ileal Amino Acid Digestibility and Apparent Metabolizable Energy, Animal Feed Science and Technology. (2015) 209, 211–218, 10.1016/j.anifeedsci.2015.08.006, 2-s2.0-84952637881.

[51] Diener S. , Solano N. M. S. , Gutierrez F. R. , Zurbrugg C. , and Tockner K. , Biological Treatment of Municipal Organic Waste Using Black Soldier Fly Larvae, Waste and Biomass Valorization. (2011) 2, no. 4, 357–363, 10.1007/s12649-011-9079-1, 2-s2.0-84856299594.

[52] Liland N. S. , Biancarosa I. , and Araujo P. , et al.Modulation of Nutrient Composition of Black Soldier Fly (*Hermetia illucens*) Larvae by Feeding Seaweed-Enriched Media, PLoS ONE. (2017) 12, no. 8, 10.1371/journal.pone.0183188, 2-s2.0-85028382864, e0183188. 28837591 PMC5570497

[53] National Research Council (NRC) , Nutrient Requirements of Fish, 1993, National Research Council, National Academy Press, Washington DC, 10.17226/2115.

[54] Lock E. R. , Arsiwalla T. , and Waagbø R. , Insect Larvae Meal as an Alternative Source of Nutrients in the Diet of Atlantic Salmon (*Salmo salar*) Postsmolt, Aquaculture Nutrition. (2016) 22, no. 6, 1202–1213, 10.1111/anu.12343, 2-s2.0-84938863515.

[55] Renna M. , Schiavone A. , and Gai F. , et al.Evaluation of the Suitability of a Partially Defatted Black Soldier Fly (*Hermetia illucens* L.) Larvae Meal as Ingredient for Rainbow Trout (*Oncorhynchus mykiss* Walbaum) Diets, Journal of Animal Science and Biotechnology. (2017) 8, no. 1, 957–969, 10.1186/s40104-017-0191-3, 2-s2.0-85021676323. PMC5494141 28680591

[56] Guerreiro I. , Castro C. , and Antunes B. , et al.Catching Black Soldier Fly for Meagre: Growth, Whole-Body Fatty Acid Profile and Metabolic Responses, Aquaculture. (2020) 516, 10.1016/j.aquaculture.2019.734613, 734613.

[57] Abdel-Tawaab A. A. , Shakweer M. S. , Khallaf M. A. , and Abdel-Latif H. M. R. , Effects of Black Soldier Fly (*Hermetia illucens* L.) Larvae Meal on Growth Performance, Organs-Somatic Indices, Body Composition, and Hemato-Biochemical Variables of European Sea Bass, *Dicentrarchus labrax* , Aquaculture. (2020) 522, 10.1016/j.aquaculture.2020.735136, 735136.

[58] Caimi C. , Renna M. , and Lussiana C. , et al.First Insights on Black Soldier Fly (*Hermetia illucens* L.) Larvae Meal Dietary Administration in Siberian Sturgeon (*Acipenser baerii* Brandt) Juveniles, Aquaculture. (2020) 515, 10.1016/j.aquaculture.2019.734539, 734539.

[59] Kjørsvik E. , Olsen C. , and Wold P. A. , et al.Comparison of Dietary Phospholipids and Neutral Lipids on Skeletal Development and Fatty Acid Composition in Atlantic Cod (*Gadus morhua*), Aquaculture. (2009) 294, no. 3-4, 246–255, 10.1016/j.aquaculture.2009.06.012, 2-s2.0-67749084100.

[60] Tran G. , Heuzé V. , and Makkar H. P. S. , Insects in Fish Diets, Animal Frontiers. (2015) 5, no. 2, 37–44.

[61] Watanabe T. , Kiron V. , and Satoh S. , Trace Minerals in Fish Nutrition, Aquaculture. (1997) 151, no. 1–4, 185–207, 10.1016/S0044-8486(96)01503-7, 2-s2.0-0030835589.

[62] Hamre K. , Yúfera M. , Rønnestad I. , Boglione C. , Conceição L. E. C. , and Izquierdo M. , Fish Larval Nutrition and Feed Formulation: Knowledge Gaps and Bottlenecks for Advances in Larval Rearing, Reviews in Aquaculture. (2013) 5, no. s1, S26–S58, 10.1111/j.1753-5131.2012.01086.x, 2-s2.0-84878388414.

[63] Mozanzadeh M. T. , Bahabadi M. N. , Morshedi V. , Azodi M. , Agh N. , and Gisbert E. , Weaning Strategies Affect Larval Performance in Yellowfin Seabream (*Acanthopagrus latus*), Aquaculture. (2021) 539, 10.1016/j.aquaculture.2021.736673, 736673.

[64] Teye-Gaga C. , Molnár P. I. , Mohammed E. A. H. , and Bársony P. , Utilization of Live Feeds in Fish Larviculture: A Review, Acta Agraria Debreceniensis. (2025) 2, no. 2, 103–116, 10.34101/actaagrar/2/15985.

[65] Xu H. , Ai Q. , and Mai K. , et al.Effects of Dietary Arachidonic Acid on Growth Performance, Survival, Immune Response and Tissue Fatty Acid Composition of Juvenile Japanese Seabass, *Lateolabrax japonicus* , Aquaculture. (2010) 307, no. 1-2, 75–82, 10.1016/j.aquaculture.2010.07.001, 2-s2.0-77955768069.

[66] Gapasin R. S. J. and Duray M. N. , Effects of DHA-Enriched Live Food on Growth, Survival and Incidence of Opercular Deformities in Milkfish (*Chanos chanos*), Aquaculture. (2001) 193, no. 1-2, 49–63, 10.1016/S0044-8486(00)00469-5, 2-s2.0-0035253640.

[67] Hauville M. R. , Zambonino-Infante J. L. , Bell G. , Migaud H. , and Main K. L. , Impacts of Three Different Microdiets on Florida Pompano, *Trachinotus carolinus*, Weaning Success, Growth, Fatty Acid Incorporation and Enzyme Activity, Aquaculture. (2014) 422-423, 268–276, 10.1016/j.aquaculture.2013.12.006, 2-s2.0-84892516574.

[68] Rayner T. A. , Jørgensen N. O. G. , and Blanda E. , et al.Biochemical Composition of the Promising Live Feed Tropical Calanoid Copepod *Pseudodiaptomus annandalei* (Sewell 1919) Cultured in Taiwanese Outdoor Aquaculture Ponds, Aquaculture. (2015) 441, 25–34, 10.1016/j.aquaculture.2015.01.034, 2-s2.0-84923247457.

[69] Evjemo J. O. , Reitan K. I. , and Olsen Y. , Copepods as Live Food Organisms in the Larval Rearing of Halibut Larvae (*Hippoglossus hippoglossus* L.) With Special Emphasis on the Nutritional Value, Aquaculture. (2003) 227, no. 1–4, 191–210, 10.1016/S0044-8486(03)00503-9, 2-s2.0-0242276314.

[70] Ljubobratovic U. , Kosanovic D. , and Demény F. Z. , et al.The Effect of Live and Inert Feed Treatment With *lactobacilli* on Weaning Success in Intensively Reared Pike-Perch Larvae, Aquaculture. (2020) 516, 10.1016/j.aquaculture.2019.734608, 734608.

[71] Samat N. A. , Yusoff F. M. , Rasdi N. W. , and Karim M. , Enhancement of Live Food Nutritional Status With Essential Nutrients for Improving Aquatic Animal Health: A Review, Animals. (2020) 10, no. 12, 10.3390/ani10122457, 2457. 33371528 PMC7767546

[72] Sealey W. M. , Gaylord T. G. , and Barrows F. T. , et al.Sensory Analysis of Rainbow Trout, *Oncorhynchus mykiss*, Fed Enriched Black Soldier Fly Prepupae, *Hermetia illucens* , Journal of the World Aquaculture Society. (2011) 42, no. 1, 34–45, 10.1111/j.1749-7345.2010.00441.x, 2-s2.0-79551658304.

[73] Sándor Z. J. , Banjac V. , and Vidosavljevi S. , et al.Apparent Digestibility Coefficients of Black Soldier Fly (*Hermetia illucens*), Yellow Mealworm (*Tenebrio molitor*), and Blue Bottle Fly (*Calliphora vicina*) Insects for Juvenile African Catfish Hybrids (*Clarias gariepinus* × *Heterobranchus longifilis*), Aquaculture Nutrition. (2022) 2022, 10.1155/2022/4717014, 4717014. 36860442 PMC9973197

[74] Borgogno M. , Dinnella C. , and Iaconisi V. , et al.Inclusion of *Hermetia illucens* Larvae Meal on Rainbow Trout (*Oncorhynchus mykiss*) Feed: Effect on Sensory Profile According to Static and Dynamic Evaluations, Journal of the Science of Food and Agriculture. (2017) 97, no. 10, 3402–3411, 10.1002/jsfa.8191, 2-s2.0-85011347403. 28000914

[75] Turchini G. M. , Torstensen B. E. , and Ng W.-K. , Fish Oil Replacement in Finfish Nutrition, Reviews in Aquaculture. (2009) 1, 10–57, 10.1201/9781439808634, 2-s2.0-84872274308.

[76] Hu Y. , Huang Y. , and Tang T. , et al.Effect of Partial Black Soldier Fly (*Hermetia illucens* L.) Larvae Meal Replacement of Fish Meal in Practical Diets on the Growth, Digestive Enzyme and Related Gene Expression for Rice Field Eel (*Monopterus albus*), Aquaculture Report. (2020) 17, 10.1016/j.aqrep.2020.100345, 100345.

[77] Stejskal V. , Tran H. Q. , and Prokesova M. , et al.Partially Defatted *Hermetia illucens* Larva Meal in Diet of Eurasian Perch (*Perca fluviatilis*) Juveniles, Animals. (2020) 10, no. 10, 10.3390/ani10101876, 1876. 33066664 PMC7602402

[78] Zhao J. , Pan J. , Zhang Z. , Chen Z. , Mai K. , and Zhang Y. , Fishmeal Protein Replacement by Defatted and Full-Fat Black Soldier Fly Larvae Meal in Juvenile Turbot Diet: Effects on the Growth Performance and Intestinal Microbiota, Aquaculture Nutrition. (2023) 2023, 10.1155/2023/8128141, 8128141. 37089257 PMC10115534

[79] Nephale L. E. , Moyo N. A. G. , and Rapatsa-Malatji M. M. , Utilization of an Insect-Based Diet by Herbivorous Fish (*Oreochromis mossambicus*) and Opportunistic Predator (*Clarias gariepinus*), Scientific African. (2024) 24, 10.1016/j.sciaf.2024.e02125, e02125.

[80] Lindsay G. J. H. , Seasonal Activities of Chitinase and Chitobiase in the Digestive Tract and Serum of Cod, *Gadus morhua* (L.), Journal of Fish Biology. (1987) 30, no. 4, 495–500, 10.1111/j.1095-8649.1987.tb05773.x, 2-s2.0-84985089409.

[81] Rapatsa M. M. and Moyo N. A. G. , Enzyme Activity and Histological Analysis of *Clarias gariepinus* Fed on *Imbrasia Belina* Meal Used for Partial Replacement of Fishmeal, Fish Physiology and Biochemistry. (2019) 45, no. 4, 1309–1320, 10.1007/s10695-019-00652-3, 2-s2.0-85065964342. 31089992

[82] Rust M. B. , Halver J. E. and Hardy R. W. , Nutritional Physiology, Fish Nutrition, 2002, 3rd edition, Academic Press.

[83] D’Hondt E. , Soetemans L. , and Bastiaens L. , et al.Simplified Determination of the Content and Average Degree of Acetylation of Chitin in Crude Black Soldier Fly Larvae Samples, Carbohydrate Research. (2020) 488, 10.1016/j.carres.2019.107899, 107899. 31981987

[84] Eggink K. M. , Pedersen P. B. , Lund I. , and Dalsgaard J. , Chitin Digestibility and Intestinal Exochitinase Activity in Nile Tilapia and Rainbow Trout Fed Different Black Soldier Fly Larvae Meal Size Fractions, Aquaculture Research. (2022) 53, no. 16, 5536–5546, 10.1111/are.16035.

[85] Pascon G. , Cardinaletti G. , and Daniso E. , et al.Effect of Dietary Chitin on Growth Performance, Nutrient Utilization, and Metabolic Response in Rainbow Trout (*Oncorhynchus mykiss*), Aquaculture Reports. (2024) 37, 10.1016/j.aqrep.2024.102244, 102244.

[86] Villanueva-Gutiérrez E. , Rodriguez-Armenta C. , González-Félix M. L. , and Perez-Velazquez M. , Incorporating Hydrolyzed Soy Protein or Black Soldier Fly (*Hermetia illucens*) Larvae Meal Into Feeds for *Totoaba macdonaldi* , Aquaculture. (2022) 554, 10.1016/j.aquaculture.2022.738152, 738152.

[87] Piccolo G. , Iaconisi V. , and Marono S. , et al.Effect of Tenebrio Molitor Larvae Meal on Growth Performance, In Vivo Nutrients Digestibility, Somatic and Marketable Indexes of Gilthead Sea Bream (*Sparus aurata*), Animal Feed Science and Technology. (2017) 226, 12–20, 10.1016/j.anifeedsci.2017.02.007, 2-s2.0-85017555919.

[88] Chaklader M. R. , Siddik M. A. B. , Fotedar R. , and Howieson J. , Insect Larvae, *Hermetia illucens* in Poultry By-Product Meal for Barramundi, *Lates calcarifer* Modulates Histomorphology, Immunity and Resistance to *Vibrio harveyi* , Scientific Reports. (2019) 9, no. 1, 10.1038/s41598-019-53018-3, 16703. 31723163 PMC6853975

[89] Terova G. , Rimoldi S. , Ascione C. , Gini E. , Ceccotti C. , and Gasco L. , Rainbow Trout (*Oncorhynchus mykiss*) Gut Microbiota Is Modulated by Insect Meal From *Hermetia illucens* Prepupae in the Diet, Reviews in Fish Biology and Fisheries. (2019) 29, no. 2, 465–486, 10.1007/s11160-019-09558-y, 2-s2.0-85064134994.

[90] Yamamoto F. Y. , Suehs B. A. , and Ellis M. , et al.Dietary Fishmeal Replacement by Black Soldier Fly Larvae Meals Affected Red Drum (*Sciaenops ocellatus*) Production Performance and Intestinal Microbiota Depending on What Feed Substrate the Insect Larvae Were Offered, Animal Feed Science and Technology. (2022) 283, 10.1016/j.anifeedsci.2021.115179, 115179.

[91] Nogales-Mérida S. , Gobbi P. , and Józefiak D. , et al.Insect Meals in Fish Nutrition, Reviews in Aquaculture. (2019) 11, no. 4, 1080–1103, 10.1111/raq.12281, 2-s2.0-85052929237.

[92] Tran H. Q. , Prokešová M. , and Zare M. , et al.Production Performance, Nutrient Digestibility, Serum Biochemistry, Fillet Composition, Intestinal Microbiota and Environmental Impacts of European Perch (*Perca fluviatilis*) Fed Defatted Mealworm (*Tenebrio molitor*), Aquaculture. (2022) 547, 10.1016/j.aquaculture.2021.737499, 737499.

[93] Li S. , Ji H. , Zhang B. , Zhou J. , and Yu H. , Defatted Black Soldier Fly (*Hermetia illucens*) Larvae Meal in Diets for Juvenile Jian Carp (*Cyprinus carpio* Var. Jian): Growth Performance, Antioxidant Enzyme Activities, Digestive Enzyme Activities, Intestine and Hepatopancreas Histological Structure, Aquaculture. (2017) 477, 62–70, 10.1016/j.aquaculture.2017.04.015, 2-s2.0-85018411484.

[94] Mastoraki M. , Ferrándiz P. M. , and Vardali S. C. , et al.A Comparative Study on the Effect of Fish Meal Substitution With Three Different Insect Meals on Growth, Body Composition and Metabolism of European Sea Bass (*Dicentrarchus labrax* L.), Aquaculture. (2020) 528, 10.1016/j.aquaculture.2020.735511, 735511.

[95] Nayak M. , Saha A. , Pradhan A. , Samanta M. , and Giri S. S. , Dietary Fish Oil Replacement by Linseed Oil: Effect on Growth, Nutrient Utilization, Tissue Fatty Acid Composition and Desaturase Gene Expression in Silver Barb (*Puntius gonionotus*) Fingerlings, Comparative Biochemistry and Physiology Part B: Biochemistry and Molecular Biology. (2017) 205, 1–12. 10.1016/j.cbpb.2016.11.009 27913275

[96] Xiao X. , Jin P. , and Zheng L. , et al.Effects of Black Soldier Fly (*Hermetia illucens*) Larvae Meal Protein as a Fishmeal Replacement on the Growth and Immune Index of Yellow Catfish (*Pelteobagrus fulvidraco*), Aquaculture Research. (2018) 49, no. 4, 1569–1577, 10.1111/are.13611, 2-s2.0-85040735682.

[97] Wang H. , Gao G. , and Chen J. , et al.Effects of Black Soldier Fly (*Hermetia illucens*) Larvae Meal Replacement for Fish Meal on Growth Performance, Muscle Quality, Antioxidant Status, Immune Function, and Gut Microbiota in Juvenile Southern Catfish (*Silurus meridionalis*), Antioxidants. (2025) 14, no. 11, 10.3390/antiox14111309, 1309. 41300465 PMC12649682

[98] Zarantoniello M. , Randazzo B. , and Gioacchini G. , et al.Zebrafish (*Danio rerio*) Physiological and Behavioural Responses to Insect-Based Diets: A Multidisciplinary Approach, Scientific Reports. (2020) 10, 2020 10.1038/s41598-020-67740-w, 10648. 32606335 PMC7326965

[99] Roncarati A. , Gasco L. , Parisi G. , and Terova G. , Growth Performance of Common Catfish (Ameiurus melas Raf.) Fingerlings Fed Mealworm (*Tenebrio molitor*) Diet, Journal of Insects as Food and Feed. (2015) 1, no. 3, 233–240, 10.3920/JIFF2014.0006, 2-s2.0-84995399429.

[100] Iaconisi V. , Marono S. , and Parisi G. , et al.Dietary Inclusion of *Tenebrio molitor* Larvae Meal: Effects on Growth Performance and Final Quality Traits of Blackspot Sea Bream (*Pagellus bogaraveo*), Aquaculture. (2017) 476, 49–58, 10.1016/j.aquaculture.2017.04.007, 2-s2.0-85018502155.

[101] Wang L. , Li J. , Jin J. N. , Zhu F. , Roffeis M. , and Zhang X. Z. , A Comprehensive Evaluation of Replacing Fishmeal With Housefly (*Musca domestica*) Maggot Meal in the Diet of Nile Tilapia (*Oreochromis niloticus*): Growth Performance, Flesh Quality, Innate Immunity and Water Environment, Aquaculture Nutrition. (2016) 23, 983–993, 10.1111/anu.12466, 2-s2.0-85015695823.

[102] Olaniyi C. O. and Salau B. R. , Utilization of Maggot Meal in the Nutrition of African Catfish, African Journal of Agricultural Research. (2013) 8, no. 37, 4604–4607, 10.5897/AJAR12013.7154.

[103] Tran H. Q. , von Siebentha E. W. , and Luce J.-B. , et al.A Novel Protein Source From Lesser Mealworm (*Alphitobius diaperinus*) Larvae Meal for European Perch (*Perca fluviatilis*): Investigation on Pellet Characteristics, Production Performance, Serum Biochemistry, Digestibility, Histology, Sensory and Trait of Fillet, and Environmental Indices, Aquaculture. (2024) 581, 10.1016/j.aquaculture.2023.740460, 740460.

[104] Bruni L. , Belghit I. , Lock E.-J. , Secci G. , Taiti C. , and Parisi G. , Total Replacement of Dietary Fish Meal With Black Soldier Fly (*Hermetia illucens*) Larvae Does Not Impair Physical, Chemical or Volatile Composition of Farmed Atlantic Salmon (*Salmo salar* L.), Journal of the Science of Food and Agriculture. (2020) 100, no. 3, 1038–1047, 10.1002/jsfa.10108. 31650558

[105] Kishawy A. T. Y. , Mohammed H. A. , and Zaglool A. W. , et al.Partial Defatted Black Solider Larvae Meal as a Promising Strategy to Replace Fish Meal Protein in Diet for Nile Tilapia (*Oreochromis niloticus*): Performance, Expression of Protein and Fat Transporters, and Cytokines Related Genes and Economic Efficiency, Aquaculture. (2022) 555, 10.1016/j.aquaculture.2022.738195, 738195.

[106] Taufek N. M. , Muin H. , Raji A. A. , Md Yusof H. , Alias Z. , and Razak S. A. , Potential of Field Cricket Meal (*Gryllus bimaculatus*) in the Diet of African Catfish (*Clarias gariepinus*), Journal of Applied Animal Research. (2018) 46, no. 1, 541–546, 10.1080/09712119.2017.1357560, 2-s2.0-85026538599.

[107] Ogunji J. , Summan Toor R.-U.-A. , Schulz C. , and Kloas W. , Growth Performance, Nutrient Utilization of *Nile tilapia Oreochromis niloticus* Fed Housefly Maggot Meal (Magmeal) Diets, Turkish Journal of Fisheries and Aquatic Sciences. (2008) 8, 141–147.

[108] Fasakin E. A. , Balogun A. M. , and Ajayi O. O. , Evaluation of Full-Fat and Defatted Maggot Meals in the Feeding of Clariid Catfish *Clarias gariepinus* Fingerlings, Aquaculture Research. (2003) 34, no. 9, 733–738, 10.1046/j.1365-2109.2003.00876.x, 2-s2.0-0038381955.

[109] Glencross B. D. , Booth M. , and Allan G. L. , A Feed Is Only as Good as Its Ingredients? A Review of Ingredient Evaluation Strategies for Aquaculture Feeds, Aquaculture Nutrition. (2007) 13, no. 1, 17–34, 10.1111/j.1365-2095.2007.00450.x, 2-s2.0-33846214792.

[110] Lu R. , Chen Y. , and Yu W. , et al.Defatted Black Soldier Fly (*Hermetia illucens*) Larvae Meal Can Replace Soybean Meal in Juvenile Grass Carp (*Ctenopharyngodon idellus*) Diets, Aquaculture Reports. (2020) 18, 10.1016/j.aqrep.2020.100520, 100520.

[111] Lim P. K. , Boey P. L. , and Ng W. K. , Dietary Palm Oil Level Affects Growth Performance, Protein Retention and Tissue Vitamin E Concentration of African Catfish, Clarias Gariepinus, Aquaculture. (2001) 202, 101–112.

[112] Ng W.-K. , Lim P.-K. , and Boey P.-L. , Dietary Lipid and Palm Oil Source Affect Growth, Fatty Acid Composition and Muscle α-Tocopherol Concentration of African Catfish, *Clarias gariepinus* , Aquaculture. (2003) 215, no. 1–4, 229–243, 10.1016/S0044-8486(02)00067-4, 2-s2.0-0037427670.

[113] Ng W. K. , Wang Y. , Ketchimenin P. , and Yuen K. H. , Replacement of Dietary Fish Oil With Palm Fatty Acid Distillate Elevates Tocopherol and Tocotrienol Concentrations and Increases Oxidative Stability in the Muscle of African Catfish, *Clarias gariepinus* , Aquaculture. (2004) 233, no. 1–4, 423–437, 10.1016/j.aquaculture.2003.10.013, 2-s2.0-1642403081.

[114] Babalola T. O. , Oyawale F. E. , Adejumo I. O. , and Bolu S. A. , Effects of Dietary Fish Oil Replacement by Vegetable Oil on the Serum Biochemical and Haematological Parameters of African Catfish (*Heterobranchus longifilis*), Iranian Journal of Fisheries Sciences. (2016) 15, no. 2, 775–788.

[115] Ng W.-K. , Chong C.-Y. , Wang Y. , and Romano N. , Effects of Dietary Fish and Vegetable Oils on the Growth, Tissue Fatty Acid Composition, Oxidative Stability and Vitamin E Content of Red Hybrid Tilapia and Efficacy of Using Fish Oil Finishing Diets, Aquaculture. (2013) 372–375, 97–110, 10.1016/j.aquaculture.2012.10.030, 2-s2.0-84869876383.

[116] Li F.-J. , Lin X. , Lin S.-M. , Chen W.-Y. , and Guan Y. , Effects of Dietary Fish Oil Substitution With Linseed Oil on Growth, Muscle Fatty Acid and Metabolism of Tilapia (*Oreochromis niloticus*), Aquaculture Nutrition. (2016) 22, no. 3, 499–508, 10.1111/anu.12270, 2-s2.0-84920913327.

[117] Hundal B. K. , Liland N. S. , Rosenlund G. , Bou M. , Stubhaug I. , and Sissener N. H. , Increasing Dietary *n* -6 Fatty Acids While Keeping *n* -3 Fatty Acids Stable Decreases EPA in Polar Lipids of Farmed Atlantic Salmon (*Salmo salar*), British Journal of Nutrition. (2021) 125, no. 1, 10–25, 10.1017/S0007114520002494. 32660682

[118] Li S. , Ji H. , Zhang B. , Tian J. , Zhou J. , and Yu H. , Influence of Black Soldier Fly (*Hermetia illucens*) Larvae Oil on Growth Performance, Body Composition, Tissue Fatty Acid Composition and Lipid Deposition in Juvenile Jian Carp (*Cyprinus carpio* var. Jian), Aquaculture. (2016) 465, 43–52, 10.1016/j.aquaculture.2016.08.020, 2-s2.0-84983770674.

[119] Sudha C. , Ahilan B. , Felix N. , Uma A. , Chidambaram P. , and Prabu E. , Replacement of Fish Oil With Black Soldier Fly Larvae Oil and Vegetable Oils: Effects of Growth, Whole-Body Fatty Acid Profile, Digestive Enzyme Activity, Haematobiochemical Responses and Muscle Growth-Related Gene Expression of Juvenile Striped Catfish, *Pangasianodon hypophthalmus* , Aquaculture Research. (2022) 53, no. 8, 3097–3111, 10.1111/are.15823.

[120] Babalola T. O. , Apata D. F. , Omotosho J. S. , and Adebayo M. A. , Differential Effects of Dietary Lipids on Growth Performance, Digestibility, Fatty Acid Composition and Histology of African Catfish (*Heterobranchus longifilis*) Fingerlings, Food and Nutrition Sciences. (2011) 2, no. 1, 11–21, 10.4236/fns.2011.21002.

[121] Egessa R. , Szűcs A. , and Ardó L. , et al.Evaluation of, *Hermetia illucens*, Larvae Oil as a Dietary Substitute for Fish and Vegetable Oils in African Catfish Hybrid (*Clarias gariepinus* × *Heterobranchus longifilis*), Aquaculture Nutrition. (2024) 2025, no. 1, 10.1155/anu/4693136, 4693136. PMC12331393 40777084

[122] Sudha C. , Ahilan B. , Felix N. , Uma A. , and Prabu E. , Effects of Dietary Protein Substitution of Fishmeal With Black Soldier Fly Larval Meal on Growth and Physiological Responses of Juvenile Striped Catfish, *Pangasianodon hypophthalmus* , Aquaculture Research. (2022) 53, no. 6, 2204–2217, 10.1111/are.15739.

[123] Zhou J. S. , Liu S. S. , Ji H. , and Yu H. B. , Effect of Replacing Dietary Fish Meal With Black Soldier Fly Larvae Meal on Growth and Fatty Acid Composition of Jian Carp (*Cyprinus carpio* var, Aquaculture Nutrition. (2018) 24, no. 1, 424–433, 10.1111/anu.12574, 2-s2.0-85040648812.

[124] Wang G. , Peng K. , and Hu J. , et al.Evaluation of Defatted Black Soldier Fly (*Hermetia illucens* L.) Larvae Meal as an Alternative Protein Ingredient for Juvenile Japanese Sea Bass (*Lateolabrax japonicus*) Diets, Aquaculture. (2019) 507, 144–154, 10.1016/j.aquaculture.2019.04.023, 2-s2.0-85064162540.

[125] Moutinho S. , Pedrosa R. , Magalhães R. , Oliva-Teles A. , Parisi G. , and Peres H. , Black Soldier Fly (*Hermetia illucens*) Pre-Pupae Larvae Meal in Diets for European Seabass (*Dicentrarchus labrax*) Juveniles: Effects on Liver Oxidative Status and Fillet Quality Traits during Shelf-Life, Aquaculture. (2021) 533, 10.1016/j.aquaculture.2020.736080, 736080.

[126] Busti S. , Magnani M. , and Badiani A. , et al.Effect of Different Inclusion Levels of Defatted *Hermetia illucens* Larvae Meal on Fillet Quality of Gilthead Sea Bream (*Sparus aurata*), Journal of Insects as Food and Feed. (2023) 9, no. 12, 1615–1629, 10.1163/23524588-20220110.

[127] Moutinho S. , Oliva-Teles A. , Martínez-Llorens S. , Monroig Ó. , and Peres H. , Total Fishmeal Replacement by Defatted, *Hermetia illucens*, Larvae Meal in Diets for Gilthead Seabream (*Sparus aurata*) Juveniles, Journal of Insects as Food and Feed. (2022) 8, no. 12, 1455–1468, 10.3920/JIFF2021.0195.

[128] Iaconisi V. , Secci G. , and Sabatino G. , et al.Effect of Mealworm (*Tenebrio molitor* L.) Larvae Meal on Amino Acid Composition of Gilthead Sea Bream (*Sparus aurata* L.) and Rainbow Trout (*Oncorhynchus mykiss* W.) Fillets, Aquaculture. (2019) 513, 10.1016/j.aquaculture.2019.734403, 2-s2.0-85071837130, 734403.

[129] Bell J. G. , Henderson R. J. , Tocher D. R. , and Sargent J. R. , Replacement of Dietary Fish Oil With Increasing Levels of Linseed Oil: Modification of Flesh Fatty Acid Composition in Atlantic salmon (*Salmo salar*) Using a Fish Oil Finishing Diet, Lipids. (2004) 39, 223–232. 15233400 10.1007/s11745-004-1223-5

[130] Mourente G. and Bell J. G. , Partial Replacement of Dietary Fish Oil With Blends of Vegetable Oils (Rapeseed, Linseed and Palm Oils) in Diets for European Sea Bass (*Dicentrarchus labrax* L.) Over a Long-Term Growth Study: Effects on Muscle and Liver Fatty Acid Composition and Effectiveness of a Fish Oil Finishing Diet, Comparative Biochemistry and Physiology B. (2006) 145, 389–399. 10.1016/j.cbpb.2006.08.012 17055762

[131] Tocher D. R. , Omega-3 Long-Chain Polyunsaturated Fatty Acids and Aquaculture in Perspective, Aquaculture. (2015) 449, 94–107, 10.1016/j.aquaculture.2015.01.010, 2-s2.0-84961381512.

[132] Turchini G. M. , Ng W.-K. , and Tocher D. R. Eds , Fish Oil Replacement and Alternative Lipid Sources in Aquaculture Feeds, 2010, 1st edition, CRC Press.

[133] Mourente G. , Dick J. R. , Bell J. G. , and Tocher D. R. , Effect of Partial Substitution of Dietary Fish Oil by Vegetable Oils on Desaturation and β-Oxidation of [1-^14^C] 18:3*n*−3 (LNA) and [1-^14^C] 20:5*n*−3 (EPA) in Hepatocytes and Enterocytes of European Sea Bass (*Dicentrarchus labrax* L.), Aquaculture. (2005) 248, 173–186.

[134] Tocher D. R. , Metabolism and Functions of Lipids and Fatty Acids in Teleost Fish, Reviews in Fisheries Science. (2003) 11, no. 2, 107–184, 10.1080/713610925, 2-s2.0-0346764654.

[135] Bandara T. , Bruge S. , Andersson A. , and Lau D. C. P. , Retention of Essential Fatty Acids in Fish Differs by Species, Habitat Use and Nutritional Quality of Prey, Ecology and Evolution. (2023) 13, no. 6, 10.1002/ece3.10158, e10158. 37274152 PMC10234757

[136] Péron M. , Bertrand M. , and Baranek E. , et al.N-3 Long-Chain Polyunsaturated Fatty Acids in Fish Physiology: From Aquaculture to Economic, Ecological and Public Health Challenges, Biochimie. (2025) 239, 4–18, 10.1016/j.biochi.2025.09.007. 40967332

[137] Bell J. G. , McVicar A. H. , Park M. T. , and Sargent J. R. , High Dietary Linoleic Acid Affects the Fatty Acid Compositions of Individual Phospholipids from Tissues of Atlantic Salmon (*Salmo salar*): Association with Stress Susceptibility and Cardiac Lesion, The Journal of Nutrition. (1991) 121, no. 8, 1163–1172, 10.1093/jn/121.8.1163, 2-s2.0-0026326385. 1861166

[138] Izquierdo M. S. , Obach A. , Arantzamendi L. , Montero D. , Robaina L. , and Rosenlund G. , Dietary Lipid Sources for Seabream and Seabass: Growth Performance, Tissue Composition and Flesh Quality, Aquaculture Nutrition. (2003) 9, 397–407.

[139] Trushenski J. T. , Blaufuss P. , Mulligan B. , and Laporte J. , Growth Performance and Tissue Fatty Acid Composition of Rainbow Trout Reared on Feeds Containing Fish Oil or Equal Blends of Fish Oil and Traditional or Novel Alternative Lipids, North American Journal of Aquaculture. (2011) 73, 194–203.

[140] Hossain M. S. , Fawole F. J. , Labh S. N. , Small B. C. , Overturf K. , and Kumar V. , Insect Meal Inclusion as a Novel Feed Ingredient in Soy-Based Diets Improves Performance of Rainbow Trout (*Oncorhynchus mykiss*), Aquaculture. (2021) 544, 10.1016/j.aquaculture.2021.737096.

[141] Laporte J. and Trushenski J. , Growth Performance and Tissue Fatty Acid Composition of Largemouth Bass Fed Diets Containing Fish Oil or Blends of Fish Oil and Soy-Derived Lipids, North American Journal of Aquaculture. (2011) 73, no. 4, 435–444, 10.1080/15222055.2011.623947, 2-s2.0-84977876938.

[142] Asdari R. , Aliyu-Paiko M. , Hashim R. , and Ramachandran S. , Effects of Different Dietary Lipid Sources in the Diet for *Pangasius hypophthalmus* (Sauvage, 1878) Juvenile on Growth Performance, Nutrient Utilization, Body Indices and Muscle and Liver Fatty Acid Composition, Aquaculture Nutrition. (2011) 17, no. 1, 44–53, 10.1111/j.1365-2095.2009.00705.x, 2-s2.0-78650896227.

[143] Maranga B. O. , Omolo K. M. , Kagali R. N. , Orina P. S. , and Kyule D. N. , Fatty Acid Composition of African Catfish (*Clarias gariepinus*) Fed on Black Soldier Fly Larvae (*Hermitia illucens*) Formulated Diets, Aquaculture, Fish and Fisheries. (2025) 5, no. 3, 10.1002/aff2.70078, e70078.

[144] Glencross B. D. , Exploring the Nutritional Demand for Essential Fatty Acids by Aquaculture Species, Reviews in Aquaculture. (2009) 1, no. 2, 71–124, 10.1111/j.1753-5131.2009.01006.x.

[145] Tallima H. and El Ridi R. , Arachidonic Acid: Physiological Roles and Potential Health Benefits—A Review, Journal of Advanced Research. (2018) 11, 33–41. 30034874 10.1016/j.jare.2017.11.004 PMC6052655

[146] Oliva-Teles A. , Enes P. , and Peres H. , Replacing Fishmeal and Fish Oil in Industrial Aquafeeds for Carnivorous Fish, Feed and Feeding Practices in Aquaculture, 2015, Woodhead Publishing, 203–233, 10.1016/B978-0-08-100506-4.00008-8.

[147] Rombenso A. N. , Trushenski J. T. , and Drawbridge M. , Saturated Lipids Are More Effective Than Others in Juvenile California Yellowtail Feeds—Understanding and Harnessing LC-PUFA Sparing for Fish Oil Replacement, Aquaculture. (2018) 493, 192–203, 10.1016/j.aquaculture.2018.04.040, 2-s2.0-85046676925.

[148] Rombenso A. N. , Turchini G. M. , and Trushenski J. T. , The Omega-3 Sparing Effect of Saturated Fatty Acids: A Reason to Reconsider Common Knowledge of Fish Oil Replacement, Reviews in Aquaculture. (2022) 14, no. 1, 213–217, 10.1111/raq.12593.

[149] Ackman R. G. , Nutritional Composition of Fats in Seafoods, Progress in Food & Nutrition Science. (1989) 13, no. 3-4, 161–289. 2699043

[150] Osibona A. O. , Kusemiju K. , and Akande G. R. , Fatty Acid Composition and Amino Acid Profile of Two Freshwater Species, African Catfish (*Clarias gariepinus*) and Tilapia (*Tilapia zillii*), African Journal of Food, Agriculture, Nutrition and Development. (2009) 9, no. 1, 608–621, 10.4314/ajfand.v9i1.19216.

[151] Ulbricht T. L. V. and Southgate D. A. T. , Coronary Heart Disease: Seven Dietary Factors, Lancet. (1991) 338, no. 8773, 985–992, 10.1016/0140-6736(91)91846-M, 2-s2.0-0025950886. 1681350

[152] Orsavova J. , Misurcova L. , Ambrozova J. , Vicha R. , and Mlcek J. , Fatty Acids Composition of Vegetable Oils and Its Contribution to Dietary Energy Intake and Dependence of Cardiovascular Mortality on Dietary Intake of Fatty Acids, International Journal of Molecular Sciences. (2015) 16, no. 6, 12871–12890, 10.3390/ijms160612871, 2-s2.0-84931274980. 26057750 PMC4490476

[153] Simopoulos A. P. , Evolutionary Aspects of Diet: The Omega-6/Omega-3 Ratio and the Brain, Molecular Neurobiology. (2011) 44, no. 2, 203–215, 10.1007/s12035-010-8162-0, 2-s2.0-80054860312. 21279554

[154] Sacks F. M. , Lichtenstein A. H. , and Wu J. H. Y. , et al.Dietary Fats and Cardiovascular Disease: A Presidential Advisory From the American Heart Association, Circulation. (2017) 136, no. 3, e1–e23, 10.1161/CIR.0000000000000510, 2-s2.0-85020532561. 28620111

[155] Hart T. L. , Kris-Etherton P. M. , and Petersen K. S. , Consuming Pecans as a Snack Improves Lipids/Lipoproteins and Diet Quality Compared With Usual Diet in Adults at Increased Risk of Cardiometabolic Diseases: A Randomized Controlled Trial, American Journal of Clinical Nutrition. (2025) 121, no. 4, 769–778, 10.1016/j.ajcnut.2025.01.024. 39880306

[156] Zhang X. , Ning X. , and He X. , et al.Fatty Acid Composition Analyses of Commercially Important Fish Species from the Pearl River Estuary, China, PLoS ONE. (2020) 15, no. 1, 10.1371/journal.pone.0228276, e0228276. 31999793 PMC6992182

[157] Aberoumand A. and Baesi F. , Evaluation of Fatty Acid- Related Nutritional Quality Indices in Processed and Raw (*Lethrinus lentjan*) Fish Fillets, Food Science & Nutrition. (2023) 11, no. 2, 963–971, 10.1002/fsn3.3131. 36789051 PMC9922119

[158] Fazio F. , Ferrantelli V. , Saoca C. , Giangrosso G. , and Piccione G. , Stability of Haematological Parameters in Stored Blood Samples of Rainbow Trout *Oncorhynchus mikiss* (Walbaum, 1792), Veterinární Medicína. (2017) 62, no. 7, 401–405, 10.17221/51/2017-VETMED, 2-s2.0-85026249985.

[159] Jimoh W. A. , Ayelola A. A. , Mowete I. E. , Yusef Y. O. , and Idi-Ogede I. , Aquaculture By-Product Meal as Fishmeal Replacer in African Catfish (*Clarias gariepinus*) Diet: Effect on Serum Biochemistry, Innate Immune Response and Oxidative Stress Markers, International Journal of Aquatic Biology. (2022) 10, no. 2, 119–130.

[160] Witeska M. , Kondera E. , Lugowska K. , and Bojarrski B. , Hematological Methods in Fish -Not Only for Beginners, Aquaculture. (2022) 547, 10.1016/j.aquaculture.2021.737498, 737498.

[161] Adedeji O. B. , Adeyemo O. K. , and Agbede S. A. , Effects of Diazinon on Blood Parameters in the African Catfish (*Clarias gariepinus*), African Journal of Biotechnology. (2009) 8, no. 16, 3940–3946.

[162] Adedeji O. B. and Adegbile A. F. , Comparative Haematological Parameters of the Bagrid Catfish (*Chrysichthys nigrodigitatus*) and the African Catfish (*Clarias gariepinus*) From Asejire Dam in Southwestern Nigeria, Journal of Applied Sciences Research. (2011) 7, no. 7, 1042–1046.

[163] Erhunmwunse N. and Ainerua M. , Characterization of Some Blood Parameters of African Catfish (*Clarias gariepinus*), American-Eurasian Journal of Toxicological Sciences. (2013) 5, 72–76.

[164] Jimoh J. O. , Rahmah S. , and Omitoyin B. O. , et al.Growth Performances of *Clarias gariepinus* Juveniles Fed With *Jatropha curcas* Seed Meal, Aquaculture Reports. (2024) 39, 10.1016/j.aqrep.2024.102433, 102433.

[165] Witeska M. , Anemia in Teleost Fishes, Bulletin of the European Association of Fish Pathologists. (2015) 35, no. 4, 148–160.

[166] Eggink K. M. and Dalsgaard J. , Chitin Contents in Different Black Soldier Fly (*Hermetia illucens*) Life Stages, Journal of Insects as Food and Feed. (2023) 9, no. 7, 855–864, 10.3920/JIFF2022.0142.

[167] Huyben D. , Vidakovic A. , Nyman A. , Langeland M. , Lundh T. , and Kiessling A. , Effects of Dietary Yeast Inclusion and Acute Stress on Post-Prandial Whole Blood Profiles of Dorsal Aorta-Cannulated Rainbow Trout, Fish Physiology and Biochemistry. (2017) 43, no. 2, 421–434, 10.1007/s10695-016-0297-0, 2-s2.0-84988691057. 27677483 PMC5374170

[168] Hrubec T. and Smith S. , Weiss D. and Wardrop K. , Hematology of Fishes, Schalm’s Veterinary Hematology, 2010, 6th edition, 193–209.

[169] Witeska M. , Kondera E. , and Bojarski B. , Hematological and Hematopoietic Analysis in Fish Toxicology—A Review, Animals. (2023) 13, no. 16, 10.3390/ani13162625, 2625. 37627416 PMC10451336

[170] Gasco L. , Finke M. , and van Huis A. , Can Diets Containing Insects Promote Animal Health?, Journal of Insects as Food and Feed. (2018) 4, no. 1, 1–4, 10.3920/JIFF2018.x001, 2-s2.0-85042748179.

[171] Ajani E. K. , Olanrewaju A. N. , and Kareem O. K. , Haematological and Immunological Changes in the Blood of African Catfish (*Clarias gariepinus*, Burchell 1822) Reared Under Different Sex Combinations, Hematologia. (2016) 5, no. 1, 1–5, 10.3923/hematol.2016.1.5.

[172] Manna S. K. , Das N. , and Bera A. K. , et al.Reference Haematology and Blood Biochemistry Profiles of Striped Catfish (*Pangasianodon hypophthalmus*) in Summer and Winter Seasons, Aquaculture Reports. (2021) 21, 10.1016/j.aqrep.2021.100836, 100836.

[173] Lorenz E. K. , Barone R. S. C. , França W. G. , Sabioni R. E. , Koch J. F. A. , and Cyrino J. E. P. , Performance, Hematology and Immunology of *Salminus brasiliensis* Fed Diets Containing Swine Liver Hydrolysate, Aquaculture. (2018) 483, 46–52, 10.1016/j.aquaculture.2017.09.040, 2-s2.0-85031086794.

[174] Belghit I. , Waagbø R. , Lock E.-J. , and Liland N. S. , Insect-Based Diets High in Lauric Acid Reduce Liver Lipids in Freshwater Atlantic Salmon, Aquaculture Nutrition. (2019) 25, no. 2, 343–357, 10.1111/anu.12860, 2-s2.0-85057285074.

[175] Loponte R. , Nizza S. , and Bovera F. , et al.Growth Performance, Blood Profiles and Carcass Traits of Barbary Partridge (*Alectoris barbara*) Fed Different Insect Meals (*Tenebrio molitor* and *Hermetia illucens*), Research in Veterinary Science. (2017) 115, 183–188, 10.1016/j.rvsc.2017.04.017, 2-s2.0-85018428859. 28472736

[176] Bai N. , Li Q. , Pan S. , Qi Z. , Deng W. , and Gu M. , Effect of Defatted Yellow Mealworm (*Tenebrio molitor*) on Growth Performance, Intestine, and Liver Health of Turbot (*Scophthalmus maximus*), Animal Feed Science and Technology. (2023) 302, 10.1016/j.anifeedsci.2023.115672, 115672.

[177] Weththasinghe P. , Hansen J. Ø. , Nøkland D. , Lagos L. , Rawski M. , and Øverland M. , Full-Fat Black Soldier Fly Larvae (*Hermetia illucens*) Meal and Paste in Extruded Diets for Atlantic Salmon (*Salmo salar*): Effect on Physical Pellet Quality, Nutrient Digestibility, Nutrient Utilization and Growth Performances, Aquaculture. (2021) 530, 10.1016/j.aquaculture.2020.735785, 735785.

[178] Chen Y. , Lawson R. , Shandilya U. , Chiasson M. A. , Karrow N. A. , and Huyben D. , Dietary Protein, Lipid and Insect Meal on Growth, Plasma Biochemistry and Hepatic Immune Expression of Lake Whitefish (*Coregonus clupeaformis*), Fish and Shellfish Immunology Reports. (2023) 5, 10.1016/j.fsirep.2023.100111, 100111. 37456711 PMC10339128

[179] Jimoh W. A. , Fagbenro O. A. , and Adeparusi E. O. , Cooked Sesame Meal in the Diet of African Catfish *Clarias gariepinus* (Burchell 1822): Effect on Haematology, Liver and Kidney Histology, Journal of Agricultural and Marine Sciences. (2022) 27, no. 2, 41–49.

[180] Teye-Gaga C. , Molnár P. I. , and Mohammed E. A. H. , et al.Effects of Replacing Fishmeal and Fish Oil With Bsf Larval Meal and Corn Oil on Growth Performance, Fillet Composition and Blood Biochemistry of Hybrid African Catfish *Clarias Gariepinus* × *Heterobranchus Longifilis* , Social Science Research Network. (2024) 1–40, 10.2139/ssrn.4943013.

